# NOS2/ARG1 axis and immune cell ratios as promising prognostic and predictive biomarkers for Cetuximab combined with chemotherapy in wt-KRAS human colorectal cancer

**DOI:** 10.3389/fimmu.2025.1700487

**Published:** 2026-01-07

**Authors:** Djehane Houria Mataam, Assia Galleze, Sarra Benkhelifa, Ryad Trari, Wafa Khelaifia, Said Belhadef, Sabrina Bouhara, Sonia Ait Younes, Anissa Benali, Hassan Mahfouf, Olivier Morales, Houda Belguendouz, Nadira Delhem, Chafia Touil-Boukoffa, Hayet Rafa

**Affiliations:** 1 Laboratory of Cellular and Molecular Biology, Cytokines and NO Synthases-Immunity and Pathogenesis, Faculty of Biological Science, University of Sciences and Technology (USTHB), Algiers, Algeria; 2 Oncology Service, EPH Rouiba, Algiers, Algeria; 3 Anatomical Pathology Department, Nafissa Hamoud, Algiers, Algeria; 4 French National Centre for Scientific Research, Joint Research Unit (CNRS UMR) 9020, INSERMU1277, CHU Lille, University of Lille, Lille, Haut de France, France; 5 Laser Assisted Therapies and Immunotherapies for Oncology (OncoThai), INSERM 1189 Unit, Lille, France

**Keywords:** cetuximab resistance, colorectal cancer, immune cell ratios, NOS2/ARG1 axis, prognostic biomarkers, systemic inflammation, tumor cells infiltrate

## Abstract

**Background:**

Resistance to epidermal growth factor receptor monoclonal antibodies (anti-EGFR), such as cetuximab, remains a major therapeutic challenge. Growing evidence suggests that local tumor immune cells and systemic inflammation influence therapeutic outcomes. Our study aimed to investigate the balance between nitric oxide synthase-2 (NOS2) and arginase-1 (ARG1) expression and its association with immune contexture and clinical outcome in cetuximab-treated colorectal cancer patients.

**Methods:**

100 patients with colorectal cancer (CRC) were included in this study. NOS2 and ARG1 expression and their metabolites were assessed using RT-qPCR, immunofluorescence, and biochemical assays. Tumor-infiltrating CD68+ pan-macrophages, CD163+ M2 like- macrophage, and CD8+ T cells were assessed using immunohistochemistry and immunofluorescence. Baseline complete blood counts were used to calculate systemic immune ratios, including the Neutrophil-to-Lymphocyte Ratio (NLR), Platelet-to-Lymphocyte Ratio (PLR), Monocyte-to-Lymphocyte Ratio (MLR), Systemic Immune-Inflammation Index (SII), and Systemic Inflammation Response Index (SIRI). Associations between NOS2/ARG1 profiles, systemic immune cell ratios, and treatment response were analyzed using Student’s t-test. Progression-free survival (PFS) and overall survival (OS) were estimated using Kaplan–Meier analysis.

**Results:**

NOS2 and ARG1 levels were elevated in CRC, particularly in the late stages. Low NOS2/high ARG1 expression correlated with increased CD68+ and CD163+ cell infiltration, whereas high NOS2/lowARG expression was associated with increased CD8+ cell density. Systemic inflammatory indices were higher in patients with CRC than in controls. In mCRC patients receiving cetuximab plus chemotherapy, responders had lower NLR, SII, SIRI, and ARG levels and higher NO levels than non-responders. High baseline SII, SIRI, and ARG levels predicted poorer PFS and OS, whereas elevated NO levels predicted better outcomes. Interestingly, a combined score integrating NO, ARG, SII, and SIRI indicated a higher prognostic value than individual markers in mCRC patients.

**Conclusion:**

Our study highlights the pivotal role of the NOS2/ARG1 axis in local immune infiltration, systemic inflammation, and clinical outcomes in mCRC patients receiving cetuximab. For the first time, we propose a novel combined score integrating NO, arginase, SII, and SIRI as simple, accessible, and non-invasive prognostic and predictive markers. Our findings may open new avenues for patient stratification and treatment optimization in precision oncology research.

## Introduction

Colorectal cancer is one of the most lethal cancers worldwide (1). According to GLOBOCAN 2022, CRC is the third most commonly diagnosed malignancy and the second leading cause of cancer-related mortality globally, accounting for more than 1.9 million new cases and approximately 904,019 deaths per year. Although advances have been made in the management and treatment of primary CRC, the prognosis of patients with metastatic CRC (mCRC) remains poor, with a 5-year survival rate of 14% (2, 3). Precision medicine has transformed CRC treatment by enabling a personalized approach based on the genetic profile of each patient. Genomic profiling has identified key mutations, such as those in KRAS, BRAF, and PIK3CA, which are involved in signaling pathways that regulate cell proliferation and survival. This information guides the selection of targeted therapies, improving treatment effectiveness while minimizing the side effects (4).

Anti-EGFR therapies have improved outcomes in patients with mCRC, however, resistance to these agents remains a significant challenge (5, 6). Tumor recurrence and therapeutic resistance are increasingly recognized as major determinants of poor prognosis, emphasizing the urgent need for more robust clinical tools to refine patient stratification (7). In this context, the identification of reliable biomarkers capable of predicting treatment response and survival would represent a substantial advancement toward personalized therapeutic strategies in mCRC research and care (8, 9).

It is now widely recognized that KRAS mutations are negative predictors of response to anti-EGFR therapy. However, studies have shown that patients with RAS wild-type manifest acquired resistance to treatment, indicating the involvement of alternative mechanisms in anti-EGFR resistance (10–13). There is increasing evidence that immune and inflammatory contexts, both systemic and within the tumor microenvironment (TME), play a central role in therapeutic responses and patient prognosis (1, 14).

Among the emerging factors contributing to this complex resistance landscape, the balance between NOS2 and ARG1 within the TME represents a key mechanism linking immune modulation and treatment resistance (15, 16). Within the TME, tumor-infiltrating lymphocytes (TILs) and tumor-associated macrophages (TAMs) are the two main components that have shown prognostic value (17, 18). Tumor-infiltrating CD8+ cytotoxic T lymphocytes have been consistently associated with improved prognosis and therapeutic response (19, 20). However, TAMs exhibit remarkably controversial effects due to their plasticity. M1-polarized macrophages, characterized by NOS2 expression, promote tumor suppression through pro-inflammatory activity, whereas M2-like macrophages, which often express ARG1, contribute to immune suppression, matrix remodeling, and therapeutic resistance (21, 22). The interplay between ARG1 and NOS2, enzymes that metabolize the common substrate L-arginine, is a central immunometabolic axis that regulates T cell function and macrophage polarization. ARG1 activity can suppress CD8^+^ T cell function by depleting arginine, whereas NOS2 promotes the activation of CD8^+^ T cells by enhancing their antitumor cytotoxic functions through upregulation of death receptor expression (23–25).

Additionally, at the systemic level, immune cell ratios, including the neutrophil-to-lymphocyte ratio (NLR), platelet-to-lymphocyte ratio (PLR), monocyte-to-lymphocyte ratio (MLR), systemic immune-inflammation index (SII), and systemic inflammation response index (SIRI), have emerged as accessible biomarkers with prognostic and predictive significance in CRC (26, 27). However, their predictive power alone remains limited, and their clinical utility is debatable. The integration of these systemic markers with intratumoral immune features could provide a more comprehensive and clinically actionable biomarker profile.

In this context, and consistent with our previous work demonstrating the implication of the iNOS/NO system in colitis-associated cancer and colorectal cancer, particularly in immune escape, tissue damage, and therapeutic response (28, 29), we sought to further investigate the NOS2/ARG1 axis by stratifying CRC patients according to high or low expression levels. This study aimed to evaluate the association between NOS2/ARG1 expression and systemic immune cell ratios with local tumor immune infiltrates (TILs, TAMs) and to assess their prognostic significance for response to cetuximab-based chemotherapy and patient survival. Importantly, this study offers a novel approach through the development of a simple and noninvasive combined score integrating NO, arginase, SII, and SIRI. This novel score aims to enhance patient stratification by identifying individuals who are more likely to benefit from treatment and experience favorable survival outcomes.

## Patients and methods

### Patients

In our study, 100 Algerian patients with histopathologically confirmed colorectal adenocarcinoma were enrolled and followed up prospectively and retrospectively between 2014 and 2025 at the Oncology Department of the Rouiba Hospital. The inclusion and exclusion criteria are shown in **Supplementary Material 2**. Additionally, 50 healthy participants were included as the control group. The control subjects were required to have no acute or chronic inflammatory disease, hematologic disorder, conditions capable of altering circulating cell counts, or history of cancer. They also had to refrain from recent use of medications or substances known to modify inflammatory parameters, including antibiotics, anti-inflammatory drugs, corticosteroids, immunosuppressants. Clinical responses were assessed after eight cycles. Only the 60 metastatic patients who received CAPOX + Cetuximab were included in the treatment response analysis, as this subgroup corresponded to the population eligible for anti-EGFR therapy and for whom predictive biomarkers were assessed. Tumor response was assessed using the Response Evaluation Criteria in Solid Tumors (RECIST) and categorized as partial response (PR), stable disease (SD), or progressive disease (PD). The characteristics of the study groups are shown in **Table 1**. This study was conducted in accordance with the Declaration of Helsinki (1964) and approved by the Ethics Committee of the National Agency for Research Development in Health and Life (ATRSSV) 20/PNR 2023/ATRSSV dated January 02, 2023. Written informed consent was obtained from all participants included in the study. The informed consent form temple is included in **Supplementary Material 3**.

**Table 1:** Clinicopathological and treatment characteristics of patients included in the study.

Clinicopathologic characteristics	Patients CRC
Total cases	N=100
Mean Age (range)	65 (48-85) years
Sex(%)	
Male	51
Female	49
Primary tumor localization (%)	
Left colon	47
Right colon	23
Transverse colon	10
Rectum	20
Tumor size (%)	
<5 cm	36
≥5 cm	64
Tumor differentiation	
Poor	31
Well	69
TNM stage (%)	
I, II	10
III, IV	90
Nodules	
N0	39
N+	61
Metastases (%)	
M0	40
M+	60
Localization metastases (%)	
Liver	27
Lung	23
Other	10
MSI	
Stable	48
Instable	52
Tumor markers (CEA)	
Negative	45
Positive	55
Therapy(%)	
Untreated	10
Treated: capecitabine + oxaliplatin	30
Treated: capecitabine + oxaliplatin + Cetuximab	60

### Plasma collection and PBMC isolation

Peripheral blood was collected into EDTA tubes. Plasma was separated by centrifugation at 4100 g for 10 minutes. The samples were then aliquoted and stored at -40°C until further analysis. Additionally, blood samples from healthy donors were also collected for use as controls. For peripheral blood mononuclear cell (PBMC) isolation, whole blood was diluted in a 1:1 ratio with sterile phosphate-buffered saline (PBS, pH 7.4), and processed using the Ficoll density gradient centrifugation method (Ficoll-Histopaque, density = 1.077, Sigma-Aldrich).

### Tissue samples

Tissue samples were collected from patients diagnosed with CRC who were admitted to the Oncology Service at Rouiba Public Hospital and Anatomical Pathology Department of Nafissa Hamoud Hospital. Normal colon mucosa was collected from the distal ends of the surgical margin during colectomy procedures.

### Nitric oxide measurement

Plasma nitric oxide levels were determined using the modified Griess assay as described by Touil- Boukoffa et al. (30). Briefly, 50 μL of plasma samples were incubated with 25 μL of Griess B (0.5% N-1-naphthylethylenediamine in 20% HCl), 25 μL of Griess A (5% sulfanilamide in 20% HCl), and 400 μL of distilled water. After 20 min incubation at room temperature in the dark, absorbance was measured at 543 nm, and nitrite concentration (μM) was calculated from a NaNO_2_ standard curve (0–128 μM).

### Plasma arginase activity

Arginase-1 activity was quantified using a modified protocol from Corraliza et al. (31). Briefly, 30 μL of plasma were incubated with 25 μL of 25 mM Tris–HCl (pH 7.4), 5 μL of 10 mM MnCl_2_, and 25 μL of 0.5 M L-arginine at 37 °C for 1 h. The reaction was terminated by adding 400 μL of an acidic mixture (H2SO4, H3P04, and H2O, 1:3:7 v/v). After the addition of 20 μL of 9% α- isonitrosopropiophenone, followed by heating at 95 °C for 1 h, absorbance was recorded at 540 nm, and ARG1 activity was expressed as μM urea·min^-1^·mg^-1^ protein.

### RNA extraction and real‐time quantitative polymerase chain reaction

Total RNA was extracted from the tissues of CRC patients with different stages and healthy mucosa (n= 12 per group) using the QIAGEN RNeasy kit (Qiagen) following the manufacturer’s protocol. RNA quantity and purity were measured using a NanoDrop spectrophotometer (Thermo Scientific, USA) with A260/A280 ratios of 1.8–2.1 and A260/A230 ratios >1.8. RNA integrity was verified by electrophoresis on denaturing agarose gels, showing distinct 28S and 18S rRNA bands with an approximate 2:1 ratio. Complementary DNA (cDNA) was synthesized using a Kit (Bio-Rad). Thus, quantitative polymerase chain reaction QPCR was performed on a MyiQ single-color real-time PCR detection system with SYBR green super mix (Bio-Rad). We normalized the gene expression amount to HPRT and GAPDH housekeeping genes. The expression of relative target genes is represented as fold differences and quantified with the 2−ΔΔCt method. Primer sequences for PCR amplification were summarized as follows: NOS2 Forward 5′-TGACCCTGAGCTCTTCGAAATC-3′, Reverse 5′ AGGGCGTACCACTTTAGCTCC-3′; Arginase 1 Forward 5′-TTGAGAAAGGCTGGTCTGCT-3′, Reverse 5’-CAAAGGGCAGGTCCCCATAA-3’; hGAPDH Forward 5′-GCCAAGGTCATCCATGACAACTTTGG-3′, Reverse 5′GCCTGCTTCACCACCTTCTTGATGTC-3′; hHRPT Forward 5′-CCCTGGCGTCGTGATTAG- 3′, Reverse 5′- ATGGCCTCCCATCTCCTT-3′.

### Histological analysis

Hematoxylin/eosin-stained samples were prepared from sections of 4 μm formalin-fixed, paraffin- embedded (FFPE) blocks taken from patients with colorectal adenocarcinoma and were classified according to the tumor-node-metastasis (TNM) staging classification. The sections were stained with hematoxylin and eosin (H&E). Histopathological assessment was performed based on epithelial integrity, crypt architecture, and the degree of mixed leukocyte infiltration, defined as the presence of a heterogeneous population of immune cells, including lymphocytes, macrophages, and neutrophils, within the tumor microenvironment. Photomicrographs of normal colon mucosa as well as tissue sections of CRC patients were examined using a light microscope at ×100 and ×400 magnifications (scale bar: 50 μm).

### Immunohistochemistry

Immunohistochemical reactions were performed on 4 μm thick slides from FFPE samples. Initially, the tissues were deparaffinized and rehydrated using a graded alcohol series. Endogenous peroxidase activity was blocked with 3% hydrogen peroxide for 10 min and nonspecific binding was blocked by incubation for 2 h in PBS containing 5% skim milk. The monoclonal mouse anti-CD68 (Sigma Aldrich, clone Kp-1, diluted 1:100) and anti-CD163 (Sigma Aldrich, clone MRQ-26, diluted 1:100) antibody (mAb) were subsequently incubated overnight at 4 °C. anti-CD68 and anti- CD163 were detected with biotinylated rabbit anti-mouse immunoglobulin horseradish peroxidase (HRP) conjugated streptavidin (1:500). Immunoreactive complexes were detected using the DAB system (Invitrogen-Life Technologies, USA). Slides were counterstained briefly with hematoxylin (Sigma Aldrich) and mounted in Eukit (Sigma Aldrich). Images were captured using a digital camera at ×100 magnification (scale bar: 500 μm). Negative control procedures included omitting the primary antibody. Positive controls were human spleen and tonsil tissues. Image acquisition, quantitative analysis, and counting of positive cells/mm2 were performed using ImageJ software with standardized settings applied uniformly across all samples. The slides were assessed in a fully blinded manner.

### Immunofluorescence staining

Tissue sections, each 4 μm thick, underwent deparaffinization with xylene and were rehydrated through a series of ethanol concentrations after being heated at 60 °C for one hour. The slides were rinsed three times with 0.1% PBS-Tween, permeabilized with 0.1% Triton-X 100 for 30 min, blocked using 3% Bovine Serum Albumin (BSA) for 2 h, and then incubated overnight at 4 °C with primary rabbit IgG antibodies against NOS2 (Invitrogen, PA3-030A, 1:250 dilution), ARG1(Invitrogen, PA5-29645, 1/500 dilution), and CD8 (Invitrogen, PA5-88265, 1/100 dilution). Goat anti-rabbit antibody conjugated with fluorescein isothiocyanate (FITC) (1:1000; Life Technologies, Carlsbad, CA, USA) was used as a secondary antibody. Finally, the slides were mounted using glycerol, and observations were performed using fluorescence microscopy at a magnification of ×200. A negative control, in which the primary antibody was omitted, was systematically included. Image acquisition and quantitative analysis and number of positive cells/mm2 were performed using ImageJ software, with standardized settings applied uniformly across all samples. Slides were assessed in a fully blinded manner.

### Data collection

Clinicopathologic characteristics and baseline counts of neutrophils, monocytes, lymphocytes, and platelets were collected from routine pre-treatment blood tests, based on patients’ medical records. Immune cell ratios and inflammatory indexes were calculated using the following formulas:

NLR= Neutrophil count/absolute lymphocyte count.
PLR= Absolute platelet count/absolute lymphocyte count.
MLR= Absolute monocyte count/absolute lymphocyte count.
SII= Platelet count × neutrophil count/lymphocyte count.
SIRI= Neutrophil count × monocyte count/lymphocyte count.

### Follow-up patients

Progression-free survival (PFS) was measured as the time between treatment initiation and disease progression or recurrence. Overall survival (OS) was defined as the time between treatment initiation and death from any cause or the date of last follow-up.

### Statistical analysis

Our data were expressed as the mean ± standard error of the mean (SEM) and analyzed statistically using Student’s t-test or one-way analysis of variance (ANOVA) test, which is appropriate for multiple comparisons. Multiple statistical comparisons were adjusted using sidak correction to control the family wise error rate. The results were considered significant when the *p*-value was less than 0.05. The normality and homogeneity of the distributions were evaluated using the Shapiro-Wilk and Fisher tests, respectively. The sample size required for the study was estimated to ensure 80% statistical power with a two-sided significance level of α = 0.05. Power calculations were performed using MedCalc Statistical Software (version 15.8, MedCalc Software, Ostend, Belgium) and G*Power software (version 3.1). The estimation was based on the expected effect size and event rate associated with the primary endpoint. The optimal cutoff values for the biomarkers were determined using receiver operating characteristic (ROC) curve analysis, with treatment response (responders vs. non-responders) as the binary outcome. The threshold corresponding to the highest Youden’s index (J = sensitivity + specificity -1) was selected. ROC curves, area under the curve (AUC), sensitivity, specificity, and Youden index were calculated. Patients were subsequently classified into “high” and “low” biomarker groups according to the derived cutoff value for all subsequent analyses. The Kaplan–Meier method was applied to progression free survival and overall survival, while survival differences between groups, defined by the established cutoff points, were compared using the log-rank test. Univariate analyses were conducted using the Cox proportional hazards model, and hazard ratios (HRs) and 95% confidence intervals (CIs) were calculated for each factor. Backward selection was then used to retain only the significant variables in the multivariate model. Multicollinearity for multivariate models was assessed using variance inflation factors (FIVs) and tolerance, a VIF < 5 and tolerance > 0.2 were considered indicative of acceptable collinearity. Statistical analyses were performed using GraphPad Prism 9 and SPSS (version 27).

## Results

### NOS2/ARG1 Axis correlates with tumor progression in sporadic colorectal cancer

Previous studies have highlighted the dysregulation of the L-Arginine nitric oxide pathway as a critical feature in colorectal cancer, promoting tumor progression, immune suppression, metastasis, and resistance to therapy (32, 33). This metabolic axis plays a central role in shaping the TME by modulating immune cell function and inflammatory responses (32, 33). Thus, considering our previous study reporting the impact of the iNOS/NO system on CRC immune escape, tissue damage, and response to therapy (29), we sought to assess the NOS2/ARG1 Axis in different stages of CRC.

**Figure 1:**
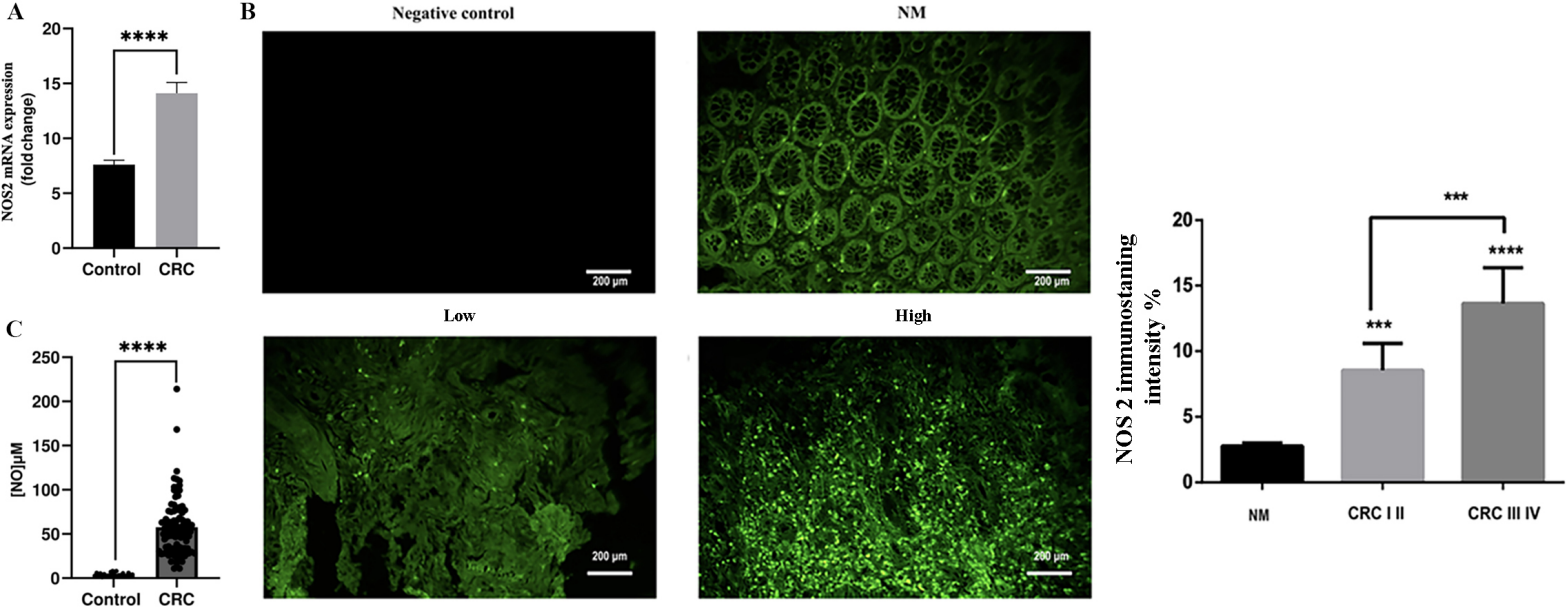
Analysis of NOS2/NO expression in control and patients with colorectal cancer. **(A)** NOS2 mRNA expression, **(B)** Representative images displaying low or high NOS2 and normal mucosa, along with quantification of NOS2 expression levels, **(C)** Plasma nitric oxide levels. NOS2 expression was quantified using the Fiji software. Data are presented as mean ± SEM. ***p < 0.001, ****p < 0.0001. (GR ×200 magnification; scale bar: 200 μm).

First, our study demonstrated that NOS2/NO expression at the mRNA (**Figure 1A**), protein (**Figure 1B**), and plasma levels (**Figure 1C**) was significantly higher in patients with colorectal cancer than in controls (*p* < 0.0001). Notably, the tissue expression of NOS2 was significantly higher in patients with late-stage disease (stages III–IV) than in those with early-stage disease (stages I–II) (*P* < 0.001).

**Figure 2:**
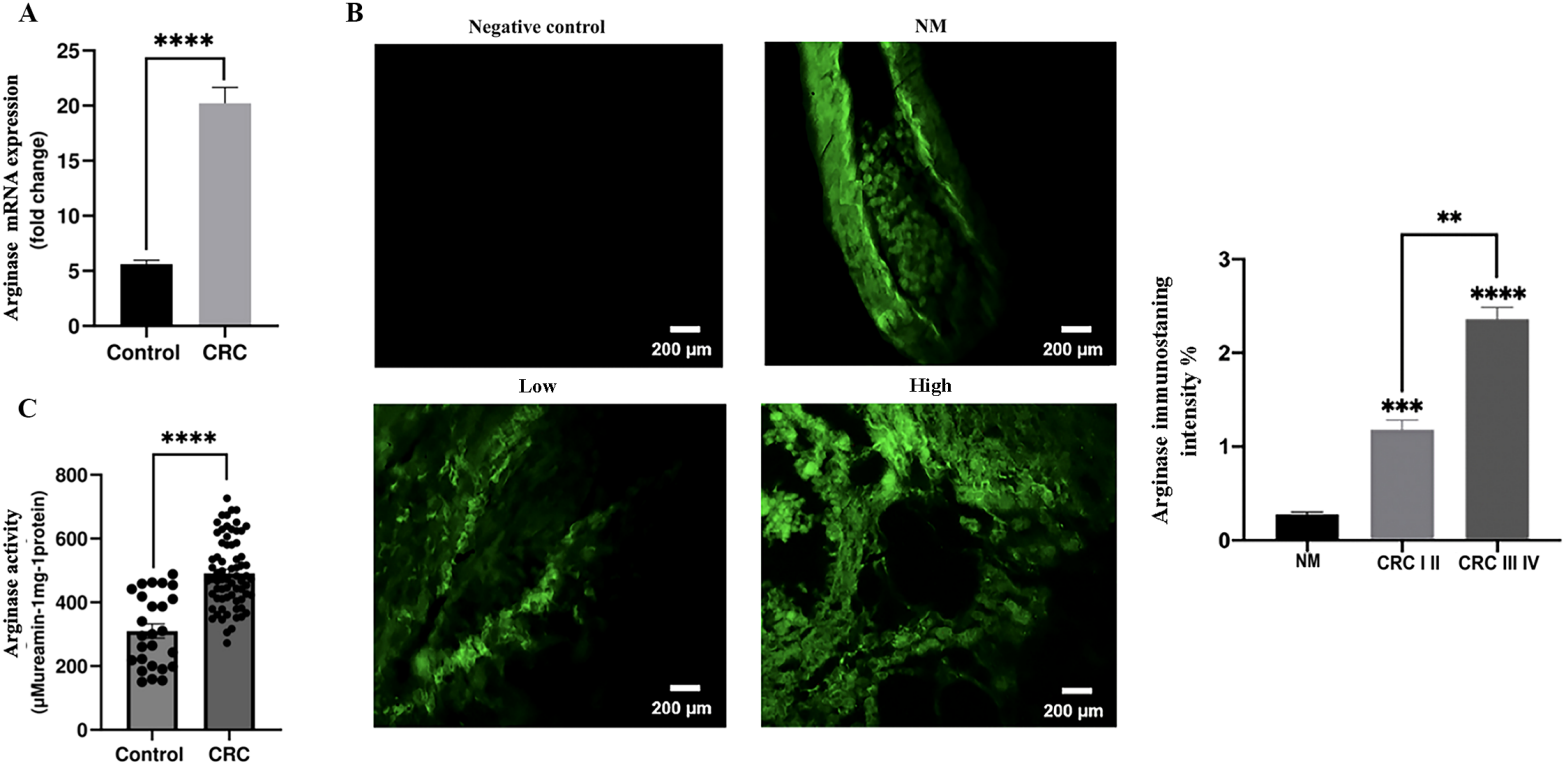
Analysis of arginase expression in control and colorectal cancer patients. **(A)** Arginase mRNA expression, **(B)** Representative images displaying low or high ARG in tumor mucosa and normal mucosa, along with quantification of protein expression levels, **(C)** Plasma arginase levels. Arginase expression was quantified using Fiji software. Data are presented as mean ± SEM. **p < 0.01, ***p < 0.001, ****p < 0.0001. (GR ×200 magnification; scale bar: 200 μm).

Second, our results also demonstrated that arginase expression at the mRNA level (**Figure 2A**), protein level (**Figure 2B**), and in plasma (**Figure 2C**) was significantly higher in patients with colorectal cancer than in controls (*p* < 0.0001). In addition, tissue arginase levels were significantly higher in late-stage cancers (stages III–IV) than in early-stage tumors (stages I–II) (*p* < 0.01). Furthermore, arginase levels were substantially elevated in cancer patients compared with those in healthy controls (CRC I, II *p* < 0.001 and CRC III, IV *p* < 0.0001). Our findings highlight the potential role of NOS2/ARG1 balance dysregulation in CRC progression.

### Tissue damages and leukocyte infiltration in CRC tissue: Relationship with NOS2/ARG1 expression

Considering the dysregulation of the NOS2/ARG1 balance assessed *in vivo* and *in situ*, we investigated the relationship between NOS2 and ARG1 expression, tissue damage, and leukocyte infiltration during CRC.

**Figure 3:**
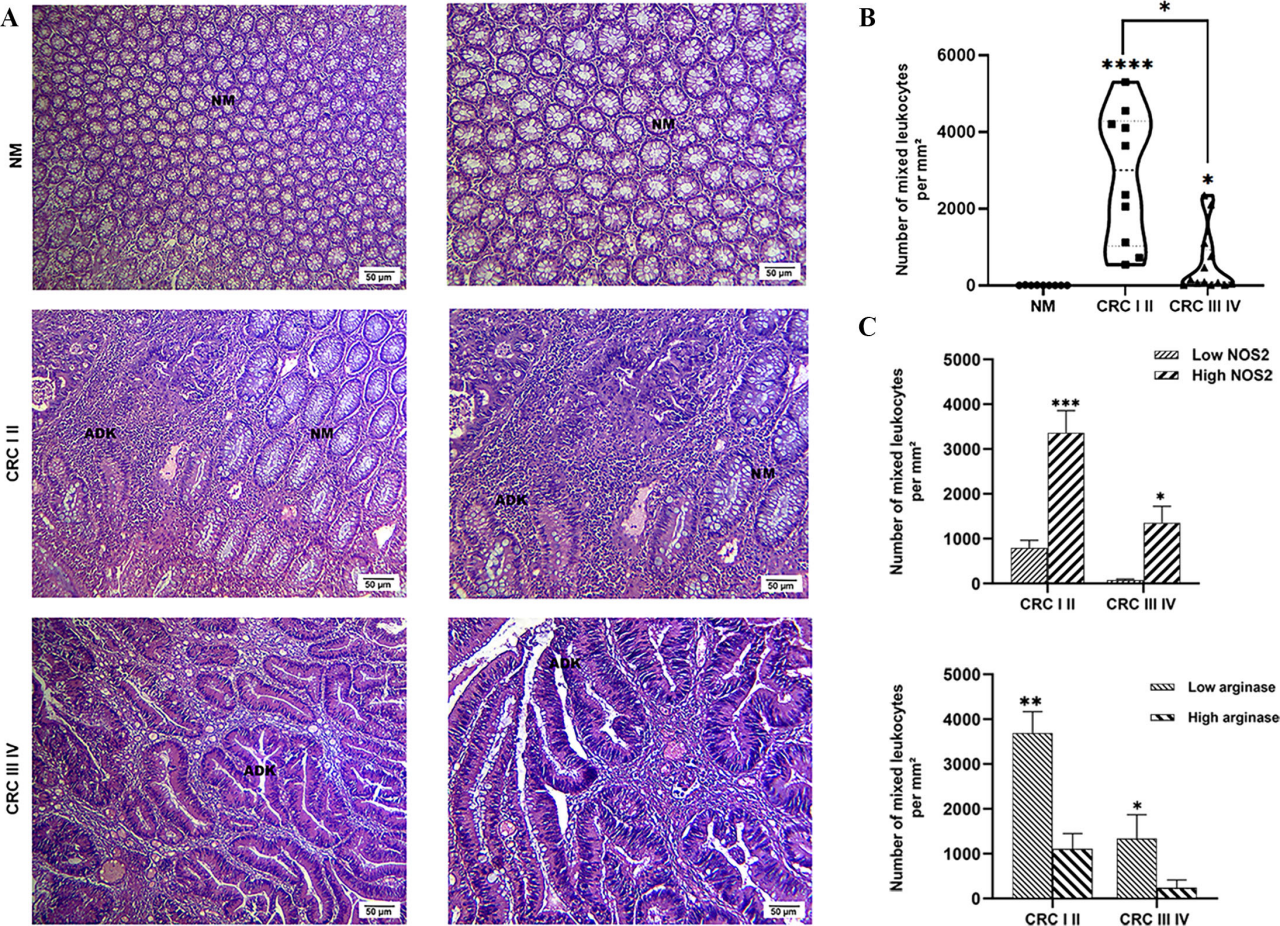
Histological analysis of normal mucosa and CRC was performed using hematoxylin and eosin staining. **(A)** Representative images of normal mucosa, early-stage CRC (CRC I and II), and late-stage CRC (CRC III and IV) at original magnification of ×100 and ×400, respectively. Scale bar: 50 μm. **(B)** Number of mixed leukocytes. **(C)** Number of leukocytes mixed in areas with high or low NOS2 and ARG expression. Data are presented as mean ± SEM. (*p < 0.05, *p* < 0.01, *p < 0.001, ****p < 0.0001).

Hematoxylin and eosin staining revealed that the healthy colon mucosa exhibited an intact structure without any histological alterations (**Figure 3A**). However, histological analysis of CRC tissues revealed severe tissue damage with significant crypt destruction and orientation, which notably increased with tumor progression (**Figure 3A**). Mixed leukocyte infiltration within tumor tissues was significantly higher in patients with early-stage CRC than in those with late-stage disease and in the control group (*p* < 0.05 and *p* < 0.0001, respectively) (**Figure 3B**). Additionally, patients with late- stage CRC exhibited significantly greater leukocyte infiltration compared to controls (*p* < 0.05) (**Figure 3B**). Interestingly, areas with high NOS2 expression were associated with significantly increased leukocyte infiltration in both early- and late-stage CRC (*p* < 0.001 and *p* < 0.05, respectively) (**Figure 3C**). Conversely, regions with high arginase expression showed significantly lower leukocyte infiltration in both early- and late-stage CRC (*p* < 0.01 and *p* < 0.05, respectively) (**Figure 3C**). These findings highlight the involvement of the NOS2/ARG system in tissue alterations and leukocyte infiltration during CRC progression.

### Immune cells infiltration in CRC tissue: Relationship with NOS2/ARG1 expression

Given the strong association between the NOS2/ARG axis and leukocyte infiltration, we hypothesized that the NOS2/ARG axis may contribute to tumor progression by influencing the composition and polarization of infiltrating immune cells. To explore this hypothesis, we examined the expression of CD68 and CD163, which are commonly associated with the immunosuppressive and pro-tumoral M2 macrophage phenotype (21, 22), as well as CD8, a marker of cytotoxic anti-tumoral T lymphocytes (19, 20).

**Figure 4:**
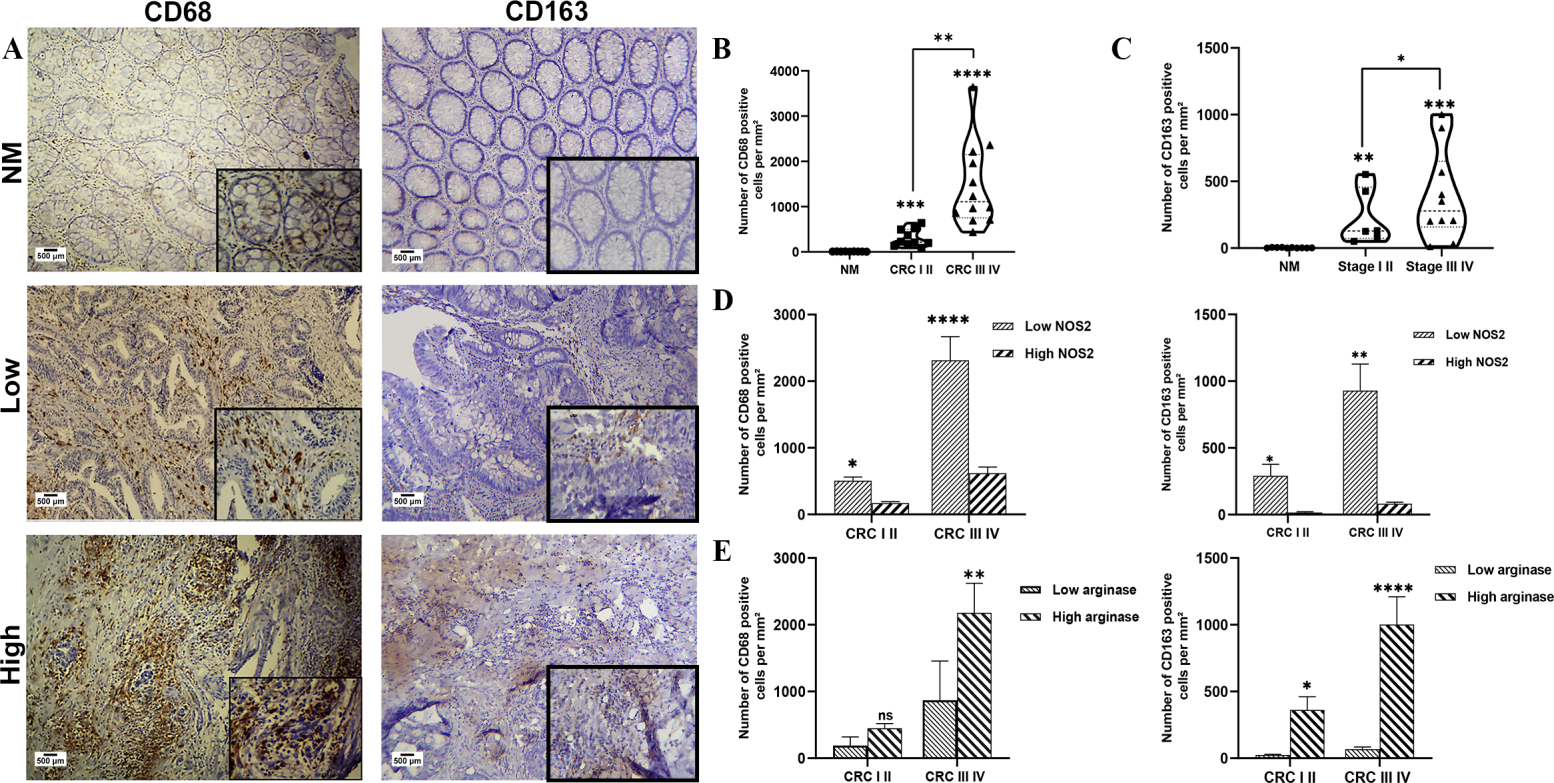
Analysis of the number of CD68^+^ and CD163^+^ cells in normal mucosa and colorectal cancer. **(A)** Representative images displaying low or high expression of CD68^+^ and CD163^+^ cells in tumor mucosa and normal mucosa. **(B)** Number of CD68^+^ cells at different stages. **(C)** Number of CD163^+^ cells at different stages. **(D)** Number of CD68^+^ and CD163^+^ cells in areas with high or low NOS2 **(E)** Number of CD68^+^ and CD163^+^ cells in areas with high or low ARG1 expression. The number of CD68^+^ and CD163^+^ TAMs infiltrating the TME was quantified using Fiji software. Data are presented as mean ± SEM (*p < 0.05, *p* < 0.01, *p < 0.001, ****p < 0.0001). Original magnification: ×100; scale bar: 500 μm.

Interestingly, our results showed that the levels of CD68^+^ and CD163^+^ TAMs infiltrating the TME were significantly higher in patients with late-stage (III, IV) than in those with early-stage (I, II) disease (*p < 0.0001, p < 0.05*). Similarly, a significant difference was observed between the expression of CD68^+^ and CD163^+^ cells in patients and normal mucosa (CD68^+^: CRC I, II *p* < 0.001; CRC III, IV *p* < 0.0001, respectively), (CD163^+^: CRC I, II *p* < 0.01; CRC III, IV *p* < 0.001, respectively) (**Figures 4B, C**). Our analysis demonstrated that areas with high NOS2 expression were associated with significantly lower infiltration of both CD68^+^ and CD163^+^macrophages then areas with low NOS2 expression. CD68^+^ cell numbers were reduced in both early- stage (CRC I, II; *p* < 0.05) and late-stage (CRC III, IV; *p* < 0.0001) tumors, whereas CD163^+^ cell counts were similarly decreased in early- and late-stage CRC (*p* < 0.05 and *p* < 0.01, respectively) (**Figure 4D**). In contrast, areas with high arginase expression showed significantly increased infiltration of CD68^+^ macrophages in late-stage CRC (*p* < 0.01) but not in early-stage tumors (**Figure 4E**). Likewise, CD163^+^ macrophage infiltration was markedly higher in regions with elevated arginase expression in both early- (*p* < 0.05) and late-stage CRC (*p* < 0.0001) (**Figure 4E**).

**Figure 5:**
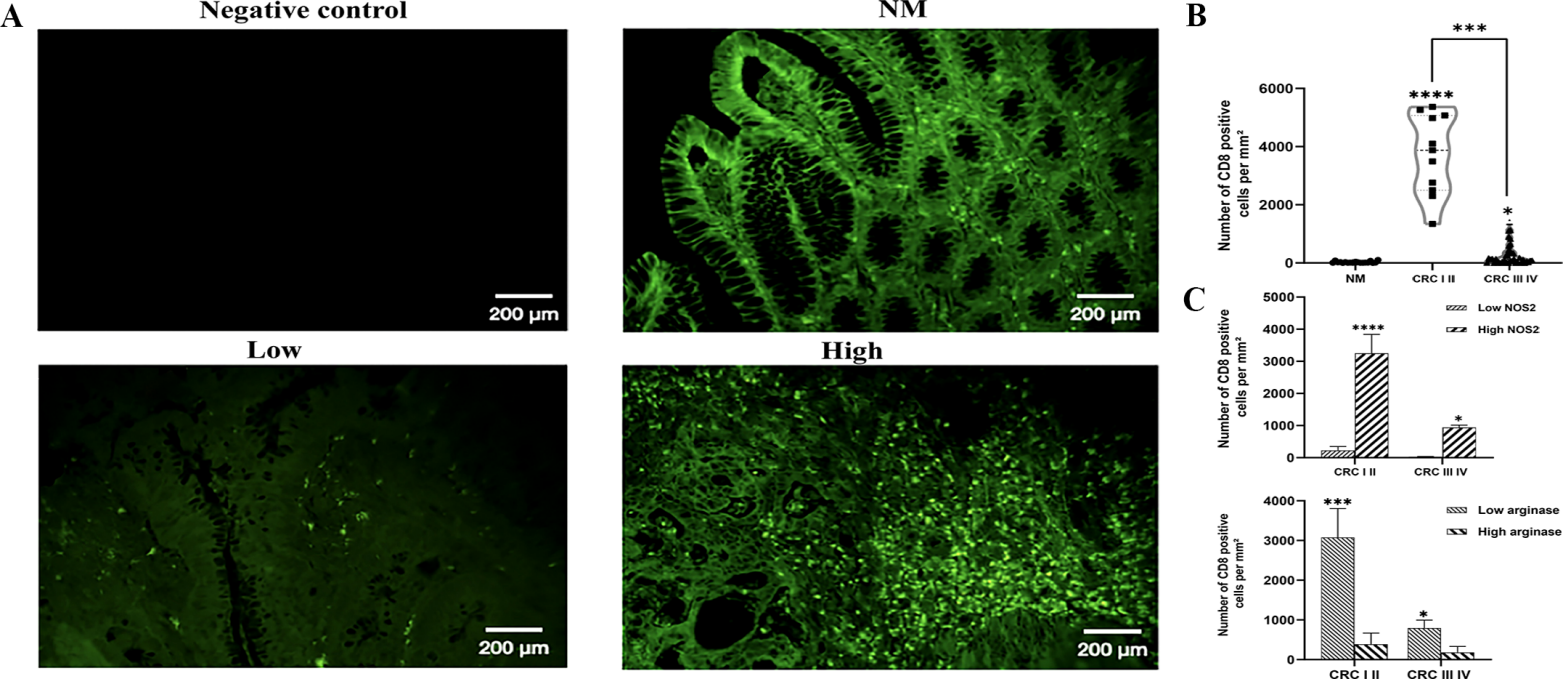
Number of CD8-positive cells per mm² in normal mucosa and colorectal cancer. **(A)** Representative images displaying low or high expression of CD8 T cell infiltrate. **(B)** Number of CD8 cell infiltration in different stages **(C)** Number of CD8 cell infiltration in areas with high or low expression of NOS2 and ARG. Quantification of CD8^+^ expression in the tumor microenvironment was analyzed using Fiji software. The results are presented as the mean ± SEM. Statistical significance: *p < 0.05, ***p < 0.001, ****p < 0.0001, one-way ANOVA. Scale bar: 200 μm; original magnification ×200.

However, the number of CD8^+^ T cells infiltrating the tumor was significantly higher in patients with early-stage colorectal cancer (Stage I–II) than in those with late-stage cancer (Stage III–IV) (*p* < 0.0001). Additionally, the difference in CD8^+^ T cell infiltration between patients and normal mucosa was significant (CRC I, II *p* < 0.0001; CRC III, IV *p* < 0.05) (**Figure 5B**). Notably, regions exhibiting elevated NOS2 expression were correlated with higher CD8^+^ T cell infiltration in both early- and late- stage CRC (*p* < 0.0001 and *p* < 0.05, respectively). In contrast, areas with higher arginase expression demonstrated significantly reduced CD8^+^ T cell infiltration at both stages (*p* < 0.001 and *p* < 0.05, respectively) (**Figure 5C**). These results suggest that the NOS2/ARG axis may critically influence tumor progression by modulating immune cell polarization and infiltration, thereby supporting the accumulation of immunosuppressive cell populations within the TME during progression.

### Relationship between NOS2/ARG1 axis and peripheral and local immune cells in CRC

**Figure 6:**
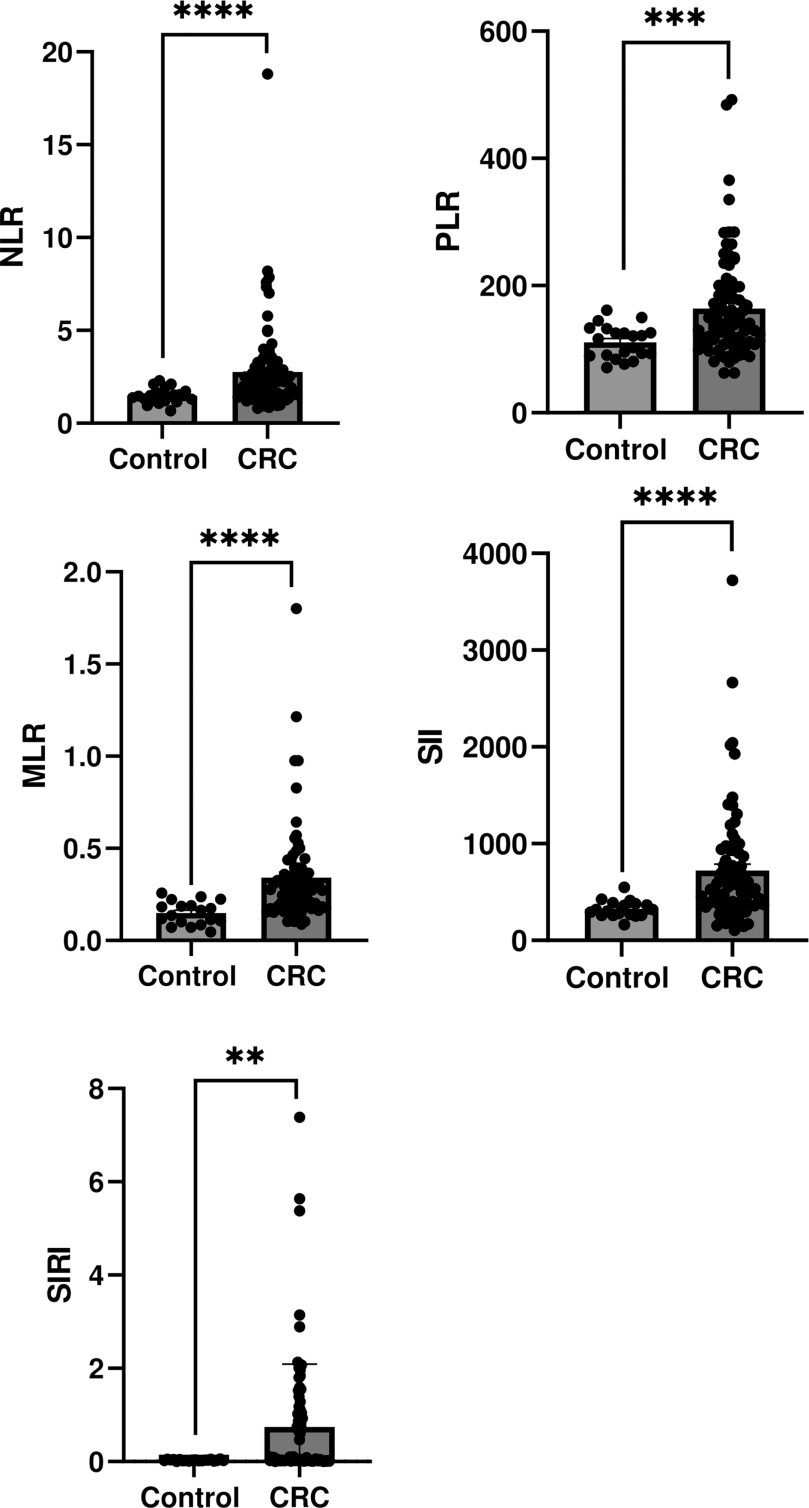
Immune cell ratios and inflammatory index levels in the control and colorectal cancer groups. **(A)** NLR, **(B)** PLR, **(C)** MLR, **(D)** SII, and **(E)** SIRI. Values are presented as mean ± SEM. **p < 0.01, ***p < 0.001, ****p < 0.0001.

To further explore the immune dynamics associated with the NOS2/ARG axis and tumor progression, we examined the ratios of circulating immune cells. These systemic immune markers may serve as peripheral blood-based surrogates for tumor-infiltrating immune cells (34), offering a less invasive and more accessible means of assessment than tissue-based methods. In particular, we focused on NLR, PLR, MLR, SII, and SIRI, which have been widely recognized as indicators of systemic inflammation and immune status in patients with cancer. Our analysis indicated a significant increase in NLR, MLR, SII *(p < 0.0001)*, PLR *(p < 0.001)*, and SIRI (*p* < 0.01) in patients with CRC compared to the controls (**Figure 6**).

**Figure 7:**
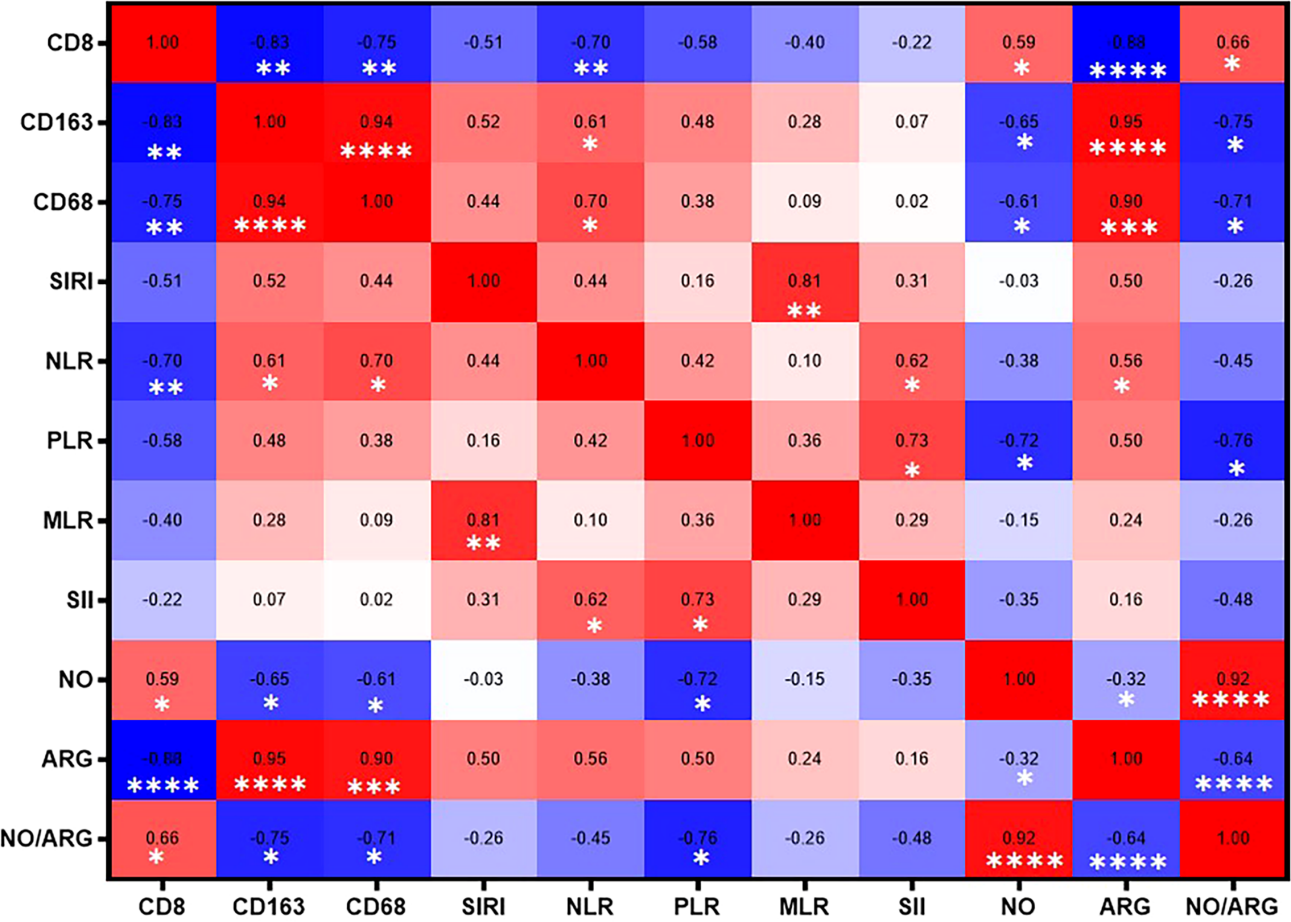
Correlation heatmap of systemic immune cell ratios, tumor cell infiltrates, and NO/ARG ratios in patients with colorectal cancer. The correlation coefficient (r) is also shown. Significant positive correlations are indicated in red, significant negative correlations in blue, and non-significant correlations are uncolored. *p < 0.05, **p < 0.01, ***p < 0.001, ****p < 0.0001.

Next, we investigated the association between systemic immune cell ratios and tumor-infiltrating immune cells in CRC, aiming to evaluate whether circulating markers reflect local immune dynamics within the tumor microenvironment. Correlation analysis revealed several significant relationships between systemic inflammatory markers and immune cell infiltration in patients with colorectal cancer. Notably, CD8^+^ T cells were significantly negatively correlated with CD163^+^ (r = −0.83, p = 0.005), CD68^+^ (r = −0.75, p = 0.015), NLR (r = −0.70, p = 0.005), and ARG (r = −0.88, p < 0.0001), while showing a moderate significant positive correlation with NO (r = 0.70, p = 0.022) and NO/ARG ratio (r = 0.70, p = 0.007). In addition, CD163^+^ infiltration was significantly positively correlated with CD68^+^ (r = 0.94, p < 0.0001) and NLR (r = 0.61, p = 0.046) and negatively correlated with NO (r = −0.65, p = 0.047) and NO/ARG ratio (r = −0.75, p = 0.017). CD68^+^ also showed a strong significant positive correlation with ARG (r = 0.90, p < 0.001) and NLR (r = 0.70, p = 0.029), and a significant positive negative correlation with NO (r = −0.61, p = 0.053) and NO/ARG ratio (r = −0.71, p = 0.025). Furthermore, strong significant correlations were observed among the systemic inflammatory indices: NLR correlated positively with SII (r = 0.61, p = 0.048) and ARG (r = 0.56, p = 0.048), and PLR correlated positively with SII (r = 0.73, p = 0.014). NO correlated negatively with ARG (r = −0.32, p = 0.030), and SIRI showed a strong positive correlation with MLR (r = 0.81, p = 0.004) (**Figure 7**). These findings suggest that the balance of the NOS2/ARG axis not only shapes the local immune cell composition but also affects peripheral immune parameters. Consequently, systemic inflammatory indices may serve as valuable non-invasive proxies for immune profiling in colorectal cancer, with potential implications for prognosis and treatment stratification.

### NOS2/ARG1 axis and peripheral immune cells as biomarkers of treatment response in metastatic colorectal cancer

Given the observed associations between the NOS2/ARG1 axis, immune cell infiltration, and systemic inflammatory markers, we next sought to evaluate the potential prognostic significance of these variables. Understanding how these immune-related parameters correlate with clinical outcomes may provide valuable insights that aid patient risk stratification and guide therapeutic decision-making. Therefore, we analyzed the relationship between these factors and treatment response.

Our findings revealed that the non-responder group exhibited significantly elevated baseline NLR, SII, SIRI, and arginase levels compared to the responder group (*p* = 0.009, *p* < 0.0001, *p* = 0.03, *p* = 0.01, respectively). Higher NO levels were observed in the responder groups than in the non- responder groups (*p* = 0.008) (**Table 2**). To further explore the prognostic relevance of these biomarkers, we constructed survival curves for each biomarker by establishing cutoff values used to stratify patients into low and high groups (**Table 3**).

**Table 2:** Comparison of baseline cell ratios and systemic inflammation indices between responders and non- responders.

Biomarker	Responder PR+SD	Non responder PD	*P*-value
NLR	1.455±0.219	3.404±0.462	**0.0091**
PLR	168.8±11.03	192.3±16.52	0.22
MLR	0.342±0.05	0.419±0.06	0.50
SII	288.9±30.25	1105±142.9	**0.0001**
SIRI	0.024±0.002	0.034±0.003	**0.03**
NO	62.83±4.65	42.62±6.60	**0.008**
Arginase	434.6±20.08	537.8±32.80	**0.01**

*p<0.05, **p<0.01, ****p<0.0001.

**Table 3:** Cut-Off values for prognostic stratification using ROC analysis.

Biomarker	AUC	Cut-off value	Sensitivity	Specificity	Youden index
NLR	0.87	2.1	59.51%	94.12%	0.5363
PLR	0.77	150.9	44.86%	94.44%	0.393
MLR	0.86	0.23	63.51%	95.24%	0.5875
SII	0.82	425.3	67.57%	100%	0.6757
SIRI	0.71	0.044	50.59%	94.44%	0.4503
NO	0.98	30	87%	100%	0.87
Arginase	0.86	461	59.14%	92.31%	0.5145

### NOS2/ARG1 expression and peripheral immune cells as prognostic and predictive markers of progression free survival and over survival in metastatic colorectal cancer

**Figure 8:**
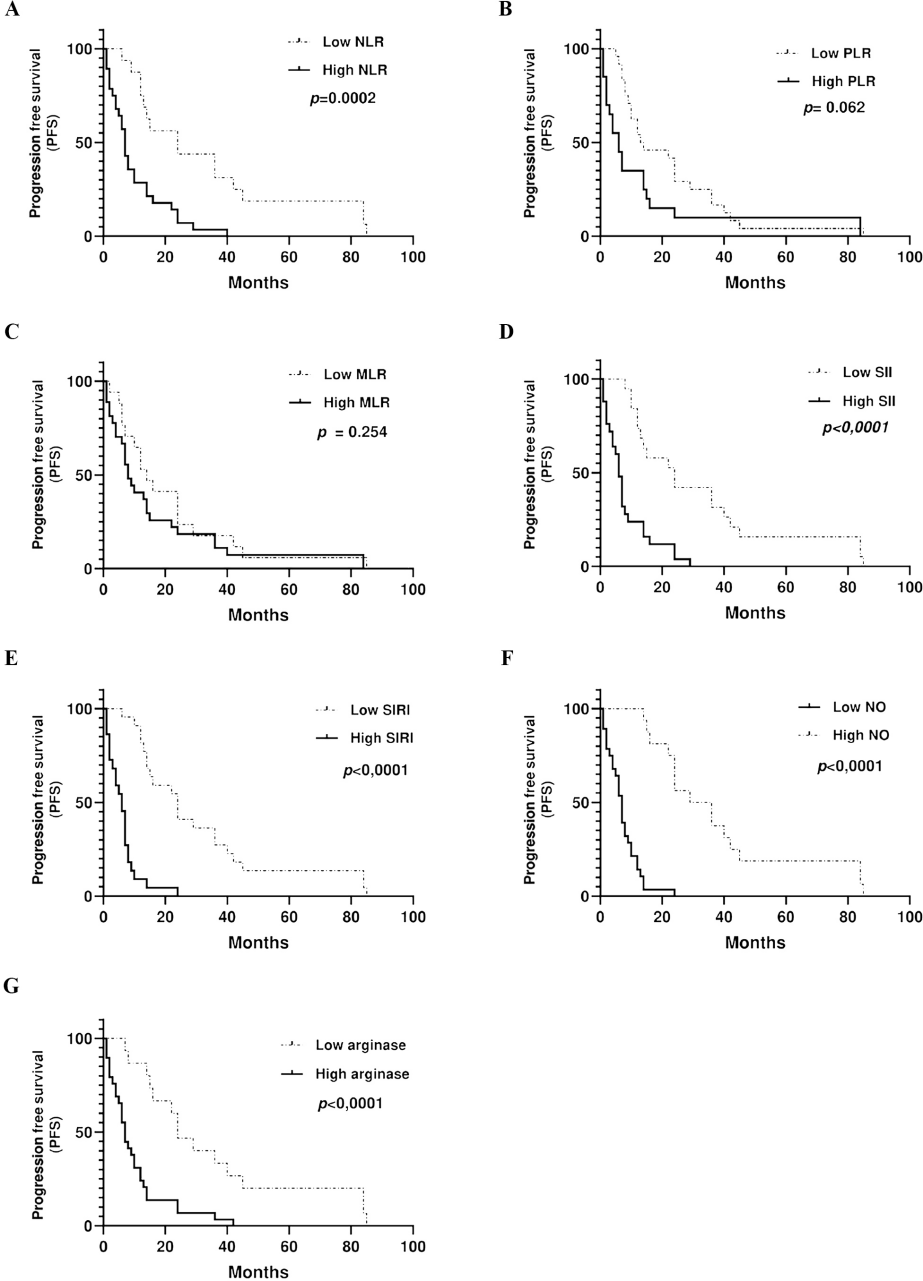
Kaplan-Meier curve of association between inflammation markers. **(A)** NLR, **(B)** PLR, **(C)** MLR, **(D)** SII, **(E)** SIRI, **(F)** NO, **(G)** arginase, and progression free survival.

**Figure 9:**
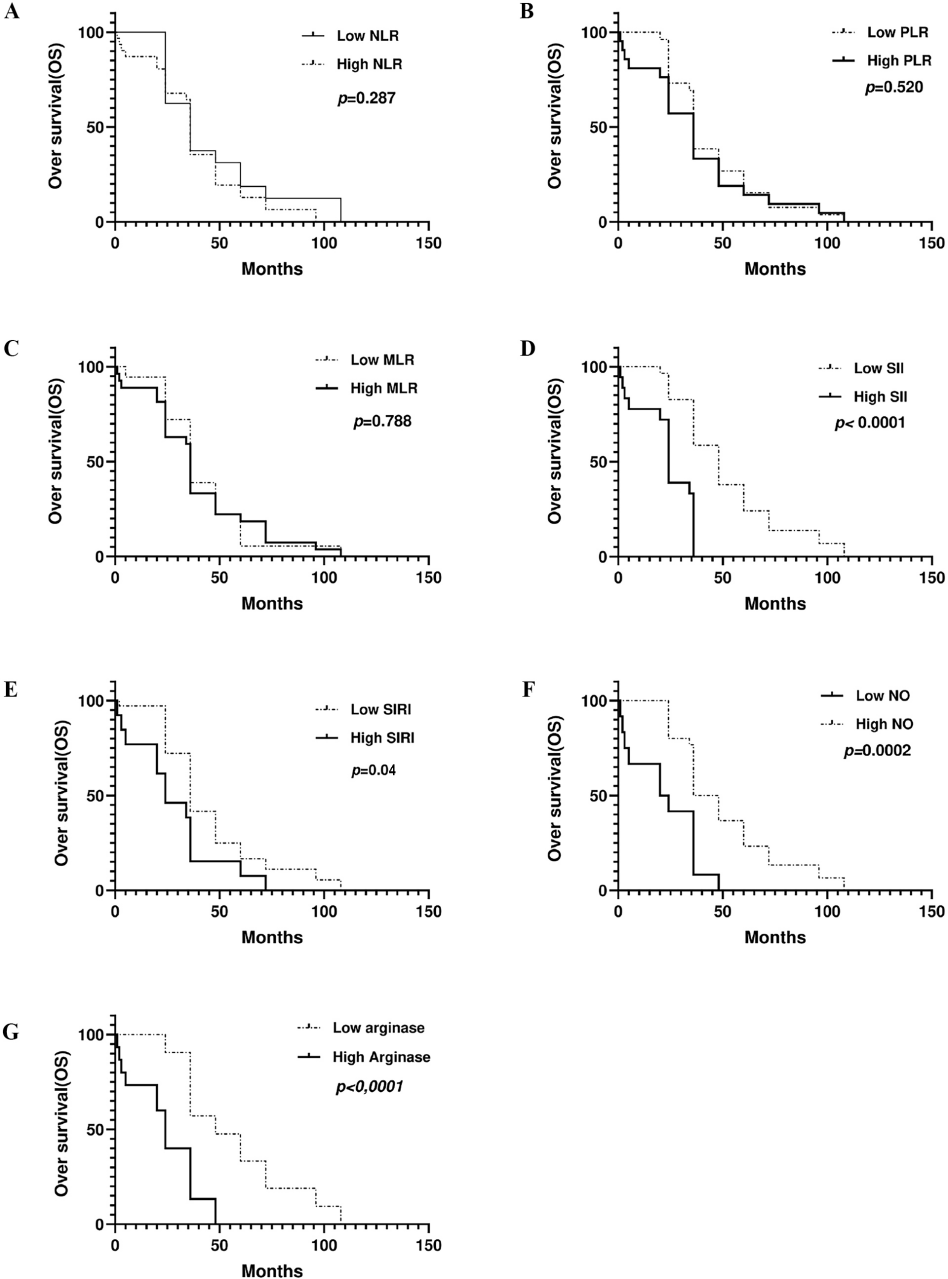
Kaplan-Meier curve of association between inflammation markers **(A)** NLR, **(B)** PLR, **(C)** MLR, **(D)** SII, **(E)** SIRI, **(F)** NO, **(G)** arginase, and survival.

Considering the interplay between systemic immune cell ratios and the tumor microenvironment, particularly their association with the NOS2/ARG1 axis and tumor-infiltrating lymphocytes, we next explored the clinical relevance of these markers. Our survival analysis revealed significant associations between inflammatory biomarkers and clinical outcomes. Patients with elevated NLR, SII, SIRI, and arginase levels exhibited significantly diminished progression-free survival (PFS) (*p* = 0.0002, *p* < 0.0001, *p* < 0.0001, and *p* < 0.0001, respectively) compared to those with low levels. In contrast, elevated NO levels were significantly associated with longer PFS (*p* < 0.0001) (**Figure 8**). Similarly, higher levels of SII, SIRI, and arginase were associated with markedly reduced overall survival (*p* < 0.0001, *p* = 0.01, *p* < 0.0001, respectively) compared with lower levels. Conversely, elevated NO levels were significantly associated with improved OS (*p* = 0.0002), highlighting their potential prognostic value (**Figure 9**).

**Figure 10:**
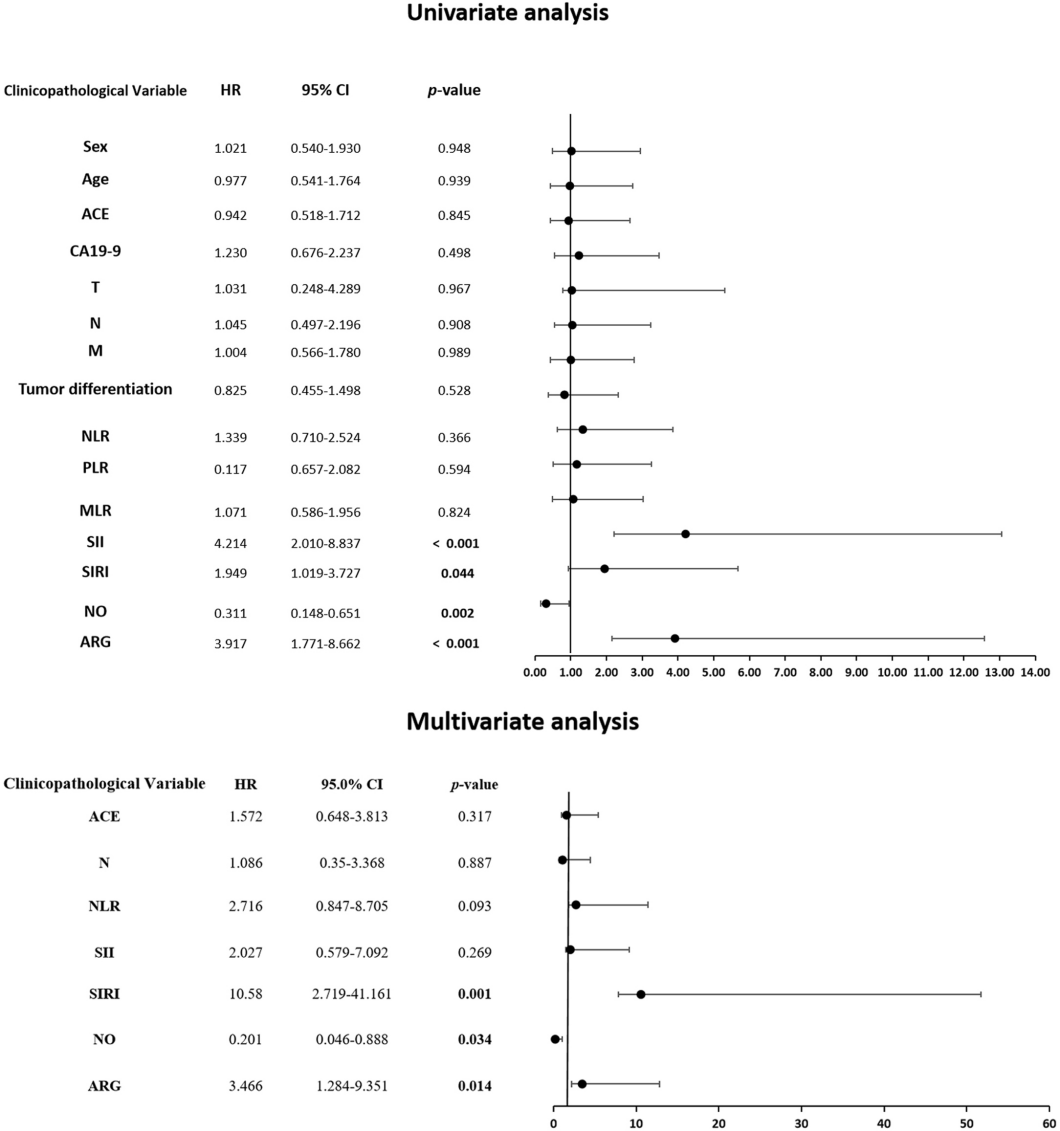
Forest plot of univariate and multivariate Cox regression analyses of PFS.

Furthermore, we sought to determine whether these inflammatory parameters could serve as independent prognostic factors. A univariate analysis revealed that elevated levels of SII (HR = 4.421, 95% CI: 2.126–9.190, *p* = 0.001), SIRI (HR = 7.058, 95% CI: 3.316–15.027, *p* < 0.001), ARG (HR = 3.786, 95% CI: 1.837–7.802, *p* < 0.001), ACE (HR = 2.241, 95% CI: 1.164–4.312, *p* = 0.016) and Node (HR = 0.291, 95% CI: 0.120 – 0.707, *p* = 0.006) had a statistically significant effect on PFS in CRC. Higher NO levels (HR = 0.086, 95% CI: 0.034–0.218, *p* = 0.0001) were associated with better survival rates. After adjusting for univariate indices, multivariate analysis was performed, and the results revealed that SIRI (HR = 10.58, 95% CI: 2.719–41.61, *p* = 0.001), arginase (HR = 10.58, 95% CI: 2.719–41.61, *p* = 0.014), and NO (HR = 3.466, 95% CI: 1.284–9.351, *p* = 0.034) remained independent prognostic factors for CRC (**Figure 10**).

**Figure 11:**
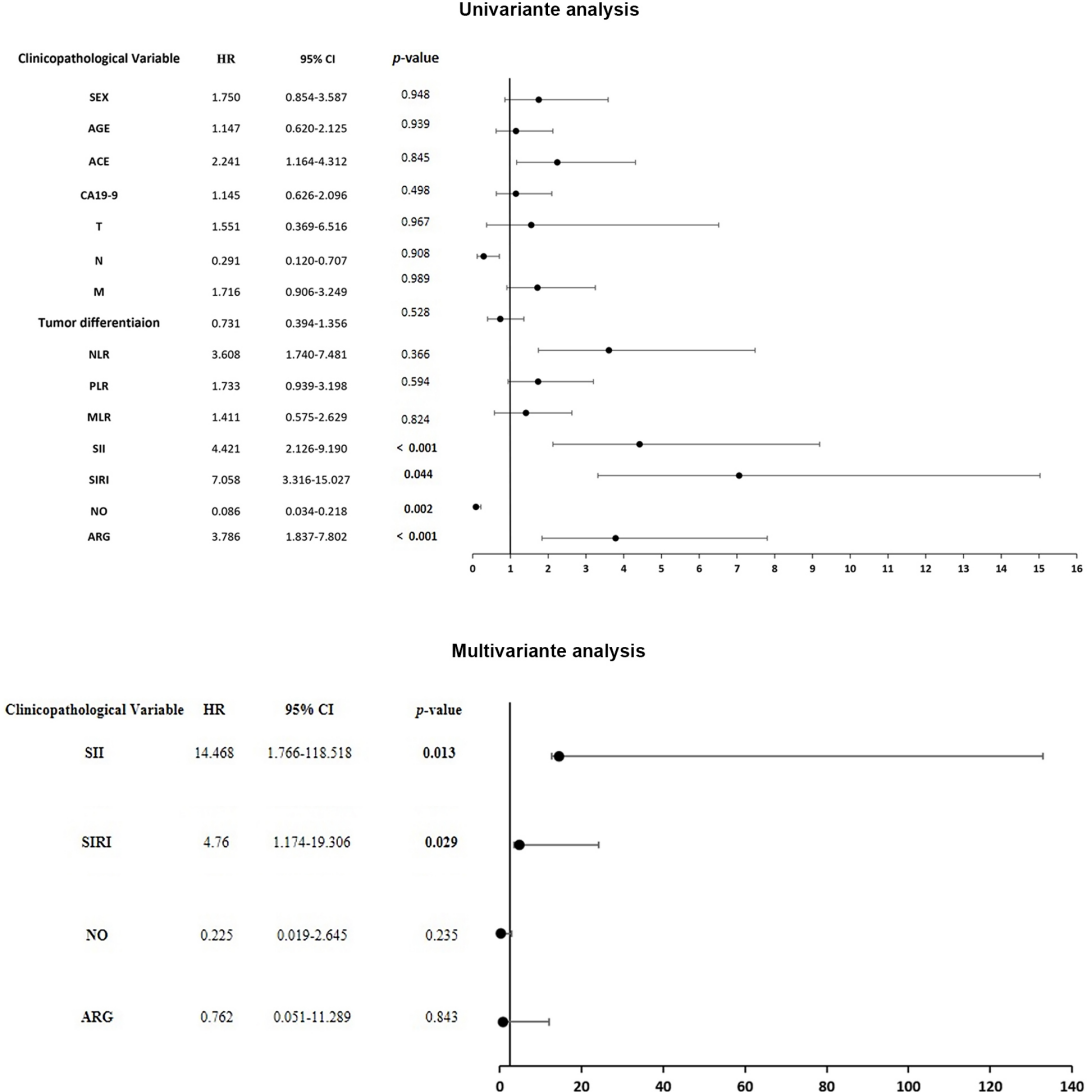
Forest plot of univariate and multivariate Cox regression analyses of OS.

Additionally, univariate analysis revealed that OS was significantly associated with several factors. Elevated SII (HR = 4.21, 95% CI: 2.01–8.84, *p* < 0.001), SIRI (HR = 1.95, 95% CI: 1.02–3.73, *p* = 0.044), and ARG (HR = 3.92, 95% CI: 1.77–8.66, *p* < 0.001) levels were linked to worse outcomes, whereas higher NO levels (HR = 0.31, 95% CI: 0.15–0.65, *p* = 0.002) were associated with better survival. In the multivariable model, after adjusting for potential confounders, both SII (HR = 14.47, 95% CI: 1.77–118.52, *p* = 0.013) and SIRI (HR = 4.76, 95% CI: 1.17–19.31, *p* = 0.029) remained significant independent prognostic markers. Other variables, including ARG and NO, were not significant in the adjusted model (**Figure 11**).

### Combined inflammatory indices score as predictors of outcome in metastatic colorectal cancer

**Figure 12:**
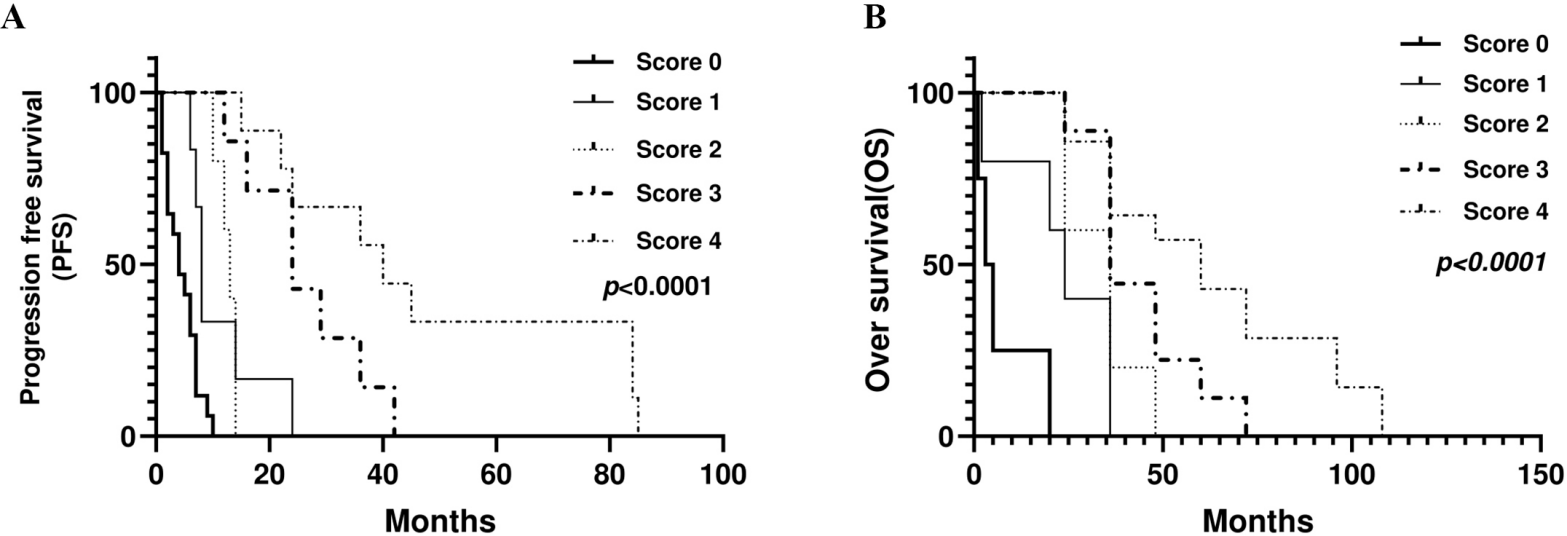
Combined inflammatory indices score as a prognostic biomarker for CRC. **(A)** PFS **(B)** OS.

Based on previous results, NO/ARG/SIRI and SII/SIRI were independently linked with PFS and OS, respectively. To enhance the prognostic value of systemic inflammatory markers in colorectal cancer, we developed a combined score integrating four widely studied indices: SII, SIRI, NO, and ARG. Each patient was assigned a score ranging from 0 to 4 based on the number of adverse markers: SII ≥ 425.3, SIRI ≥ 0.044, NO < 30, and ARG > 461. A score of 0 indicated that all markers were within the low-risk range (low SII, SIRI, and ARG and high NO levels), whereas a score of 4 indicated that all markers were within the high-risk range (high SII, SIRI, and ARG and low NO levels). Scores of 1, 2, and 3 reflected intermediate combinations of the elevated markers. Our analysis revealed that higher inflammatory scores were significantly associated with poorer progression-free survival and overall survival (p < 0.0001), suggesting that the combined score may serve as a more robust prognostic indicator than any single marker (**Figure 12**).

## Discussion

Colorectal cancer is a complex disease with multiple factors contributing to its pathogenesis (35). Previous studies have reported the relationship between NOS2/ARG1 as a key regulator of the TME, influencing immune cell infiltration and treatment response (32, 33, 36). NOS2 and ARG1 compete for the common substrate L- arginine but drive divergent immunological pathways: NOS2 promotes pro-inflammatory and cytotoxic responses which are associated with better therapeutic outcomes and improved prognosis (16, 37–39), whereas elevated arginase activity depletes L-arginine, suppresses T cell proliferation, and promotes the recruitment of immunosuppressive TAMs, which are correlated with poor prognosis (15, 40–42). In our study, we showed a significant upregulation of NOS2/NO and arginase levels in tissue and plasma compared to the control group, and these levels increased with the progression of the disease. These findings support both our earlier observations and those of other researchers showing the significant role of these enzymes in the pathophysiology of CRC and their potential as prognostic markers (28, 29, 40, 43).

Previous studies have established the NOS2/ARG1 axis as a critical modulator of tumor progression and metastasis. Wang et al. (44) reported that activation of ARG1 in the CT26 murine CRC model enhanced metastatic colonization of the liver and lungs and promoted tumor cell migration, whereas pharmacological inhibition of arginase significantly reduced these malignant features. Conversely, NOS2 plays a multifaceted role in CRC. Li et al. (45) demonstrated that elevated NOS2 expression has been shown to reduce tumorigenicity *in vivo* and inhibit CRC cell proliferation and survival by inducing apoptosis through suppression of the NF-κB signaling pathway. Additionally, Cunha et al. (46) reported that genetic deletion of NOS2 in murine T cells impaired effector differentiation, reduced tumor infiltration, and compromised recall responses and adoptive cell transfer efficacy, highlighting the essential role of endogenous nitric oxide in supporting T cell mediated immune responses within the TME. In contrast, chronic NOS2 expression can promote inflammation-driven carcinogenesis, angiogenesis, and DNA damage via sustained NO production (47).

Notably, NOS2 and ARG1 are expressed not only by tumor cells but also by immune cells that infiltrate the TME (48, 49). ARG1^+^ and NOS2^+^ immune cells were detected in the lamina propria and submucosal regions of the colon, reflecting their contribution to shaping local immune dynamics (50–53). NOS2 is expressed by multiple immune cell subsets, including T cells, macrophages, and mature dendritic cells, and modulates their function through post-translational modifications of key transcriptional and signaling proteins (54, 55). Moreover, MDSCs have been shown to be a major source of ARG1 in the tumor setting, further supporting their immunosuppressive function (56).

Given the observed upregulation of the NOS2/ARG1 axis both *in vivo* and *in situ*, we aimed to examine the relationship between NOS2 and ARG1 expression, tissue damage, and the subtypes of infiltrating immune cells during CRC progression. Our results indicate that NOS2 upregulation is associated with tissue damage and an increase in leukocyte infiltration, which occurs in a stage- dependent manner. These findings are consistent with a study conducted by Benkhelifa et al. (29). Conversely, ARG expression is associated with low leukocyte infiltration. This reduction is consistent with the fact that elevated ARG1 activity leads to the loss of T-cell receptor ζ-chain expression and induces cell-cycle arrest, resulting in T-cell exhaustion hallmarks of immune-cold tumors characterized by low tumor infiltrate (25, 57). Notably, inhibition of ARG by OATD-02, a novel arginase inhibitor, reprograms tumor metabolism by restoring L-arginine availability, thereby shifting the tumor microenvironment toward a more immunoactive state (39).

To further refine our investigation, we determined which immune cell types were affected by the NOS2/ARG1 axis. First, we assessed the expression of CD68 and CD163 in both normal and tumor mucosa. Our results showed that CD68^+^ and CD163^+^ TAMs were more abundant in the tumor mucosa than in the normal mucosa, with higher levels in late-stage CRC. This finding is consistent with those of previous studies (18, 42, 58–60). CD68 is commonly used as a pan-macrophage marker, whereas CD163 is more specifically associated with M2 macrophages (61). In colorectal cancer, M2 macrophages promote tumor progression by secreting multiple immunosuppressive factors, including IL-10, TGF-β and arginase, which suppress cytotoxic T cell activity and enhance regulatory T cell expansion (21). They also contribute to extracellular matrix remodeling, angiogenesis, and tumor cell migration through elevated production of VEGF-A, VEGF-C and MMPs (22). Conversely, M1 macrophages exhibit pro-inflammatory and antitumor activities by directly killing tumor cells through the production of reactive oxygen and nitrogen species, as well as TNF-related apoptosis- inducing ligand (TRAIL) (62). Previous studies have reported that NOS2 is upregulated in M1 macrophages and reflects pro-inflammatory activity, whereas ARG1 expression is commonly observed in association with M2-like macrophage phenotypes (42, 63).

Our data indicated that areas with low NOS2 and high arginase expression were correlated with higher CD68^+^ and CD163^+^ macrophage infiltrations. In contrast, areas with high NOS2 and low arginase expression were associated with increased CD8^+^T cell infiltration. Our results are consistent with those reported by (24, 64), suggesting that the M2 macrophage subpopulation predominates in patients with CRC undergoing progression.

The balance between M1/M2 macrophage polarization profoundly influences the infiltration and activity of CD8^+^ T lymphocytes. In our study, we observed a significantly higher infiltration of CD8^+^ T lymphocytes in patients with early-stage CRC than in those with late-stage CRC. Our results are consistent with those of Kasurinen et al. (65), who reported that reduced CD3^+^ and CD8^+^ T-cells correlated with tumor progression and an increased risk of recurrence, highlighting the critical role of the TME in disease progression. Consistent with this, a recent pancancer single-cell RNA sequencing analysis across nine tumor types revealed that distinct TAM subsets occupy functionally different niches within the TME. Pro-inflammatory TAMs were located in regions enriched with exhausted CD8^+^ T cells, whereas pro-tumorigenic TAMs were confined to hypoxic niches characterized by a T cell-excluded phenotype (66).

To further explore the immune dynamics associated with the NOS2/ARG1 axis and tumor behavior we analyzed the proportions of circulating immune cells. These systemic immune cell ratios may act as peripheral blood-based substitutes for TILs (34), providing a less invasive and more readily available method of evaluation than tissue-based approaches. Our study indicated that the immune cell ratios and inflammatory indices, including NLR, PLR, MLR, SII, and SIRI, were higher in patients with CRC than in the control group. These findings are consistent with those of previous studies on solid cancers as well as lung cancer and oral squamous cell carcinoma (67–69).

Additionally, the association between systemic biomarkers and immune cell infiltrates tumors showed that CD8^+^T-cell counts were negatively correlated with the NLR. Neutrophils, which represent the predominant subset of MDSCs, have been linked to poor prognostic outcomes in various malignancies (70). In addition, Ohashi et al. (71) reported that NLR was negatively correlated with T cell infiltration, further supporting the notion that an elevated NLR is a marker of poor prognosis in cancer. Recently, Tan et al. (72) reported that a high NLR was associated with an increase in tumor neutrophils and a decrease in CD8^+^ T-cells in TME on a penile squamous cell carcinoma.

Interestingly, CD8^+^ cell infiltration was positively correlated with plasma nitric oxide, NO/ARG ratio, and negatively correlated with the plasma arginase levels. In contrast, CD68^+^ and CD163^+^ cells were positively associated with arginase and negatively associated with nitric oxide, suggesting immune-metabolic patterns commonly observed in the tumor microenvironment.

Collectively, our findings indicate that late-stage CRC is characterized by both systemic and local immunosuppressive profiles more than early-stage CRC. In this context, we investigated the prognostic and predictive significance of the NOS2/ARG1 axis and immune cell ratios in chemotherapy combined with cetuximab in wt-KRAS metastatic colorectal cancer. Cetuximab-based therapy is widely used for treating mCRC, especially in patients with wt-RAS tumors. However, a significant proportion of patients develop resistance and fail to benefit from treatment. Therefore, it is clinically critical yet challenging to identify reliable biomarkers of resistance to optimize therapeutic strategies for each patient. Our current study demonstrated that responder CRC patients exhibited significantly lower levels of NLR, SII, SIRI, and arginase compared to the non-responder group. However, NO levels were higher in the responder group than in the non-responder group. Several recent studies have assessed the prognostic value of inflammatory indices in patients with CRC. Jiang et al. (73) documented that low pre-treatment NLR, PLR, and SII correlated with enhanced early response to cetuximab therapy. Similarly, Passardi et al. (74) reported that the SII may serve as an effective prognostic marker for patients who are candidates for first-line chemotherapy with bevacizumab in mCRC treatment.

In addition, our survival analysis demonstrated significant associations between inflammatory biomarker levels and clinical outcomes. High baseline SII, SIRI, and arginase levels were associated with poor PFS and OS. A high NLR was significantly associated with poor PFS but not OS rates. Our results are consistent with those of previous studies (59, 75–77), indicating systemic inflammation and altered immune responses, which can negatively impact patient outcomes. Additionally, Ma et al. (40) reported that elevated Arg-1 expression is closely linked to advanced CRC and may serve as a negative prognostic marker for CRC. Similarly, Qiang et al. (78) found that high ARG-1 levels were associated with poor prognosis in patients with intrahepatic cholangiocarcinoma. Consistent with these findings, Tran et al. (79) demonstrated that increased ARG1 expression is linked to poor prognosis in mouse models of endometrial cancer. More recently, Lin et al. (80) reported that ARG1 is upregulated in CRC and associated with reduced overall survival.

However, elevated NO levels were associated with improved prognostic outcomes, including longer PFS and OS. The NO prognostic model demonstrated strong potential as an independent prognostic biomarker for early onset CRC, offering a novel perspective for improving prognostic prediction in this patient population (81). Additionally, Giatromanolaki et al. (37) reported that NOS2-positive TILs may serve as potential markers of an active antineoplastic immune response. Higher NO levels may exert cytotoxic effects and induce apoptosis or programmed cell death in cancer cells and affect the apoptosis process by regulating Bcl-2 family proteins, promoting the expression of pro-apoptotic proteins such as Bax and Bad, and inhibiting the expression of anti-apoptotic proteins Bcl-2 and Bcl-xL (82).

Recently, an innovative therapeutic approach proposed by Wang et al. (83), based on engineered microparticles capable of selectively modulating arginine metabolism and reprogramming TAMs toward a pro-inflammatory phenotype using piceatannol-3′-O-glucoside, which inhibits arginase activity while activating nitric oxide synthase, effectively reduces the number of M2-like macrophages and promotes M1-like polarization. This dual action not only enhances antitumor immunity but also exerts significant inhibitory effects on tumor growth *in vivo*, highlighting a promising strategy to overcome ARG1-mediated immunosuppression. In particular, the natural compound Wang et al. (83).

Interestingly, in the multivariate analysis, SII and SIRI emerged as independent prognostic factors for PFS, whereas SII, NO, and arginase were independent predictors of OS. To enhance the prognostic value of these markers in CRC, we propose a combined score integrating four widely studied indices: NO, arginase, SII, and SIRI. To our knowledge, this is the first study to examine the value of combining these scores in the evaluation of outcomes in patients with CCR. Our results showed that the OS and PFS of patients with high combined scores were significantly lower than those of patients with low combined scores. This finding suggests that the combined score may be a more robust predictor of prognosis than any other markers. Recent studies have highlighted the predictive value of combinations of different inflammatory markers. A study on patients with hepatocellular carcinoma after liver transplantation indicated that the AUC of NLR-PLR was the largest, followed by NLR and PLR alone, which proved that the combined scoring system was more accurate than the previous single index in the prognosis of hepatocellular carcinoma (84). Similarly, Yang et al. (85) identified the pretreatment NLR-PLR score as an independent risk factor for the prognosis of non-metastatic nasopharyngeal carcinoma, demonstrating that this combined score could better distinguish patients with favorable outcomes after treatment than NLR or PLR alone. Additionally, Abudukelimu et al. (69) reported that the combined use of CA125, NSE, NLR, PLR, and SIRI offers superior diagnostic value compared to any single marker, demonstrating higher sensitivity and specificity.

## Conclusion

The current study highlights a strong association between the NOS2/ARG1 axis and both local and systemic immune contexture, as well as clinical outcomes in patients with mCRC treated with cetuximab combined with chemotherapy. Higher NOS2 and lower arginase levels were associated with increased CD8^+^ infiltration, whereas low NOS2 and high arginase levels correlated with elevated densities of CD68^+^ and CD163^+^ cells. Clinically, patients with high NOS2 and low ARG, NLR, SII, and SIRI levels exhibited better outcomes, longer PFS and OS, whereas the opposite profile predicted poor therapeutic response and unfavorable survival outcomes. Furthermore, we propose, for the first time, a novel combined score integrating the NO, arginase, SII and SIRI as a simple, accessible, and non-invasive prognostic and predictive tool. This combined score could stratify patients into distinct prognostic groups, with high scores identifying those more likely to respond to treatment and achieve longer PFS and OS, and low scores indicating poor therapeutic benefit and worse survival outcomes. However, validation of these findings in larger cohorts of mCRC patients is needed to highlight the role of this novel combined score in predicting clinical outcomes in routine clinical practice.

## Data Availability

The original contributions presented in the study are included in the article/**Supplementary Material**. Further inquiries can be directed to the corresponding authors.

## References

[1] Siegel RL Giaquinto AN Jemal A . Cancer statistic. CA: A Cancer J Clin. (2024) 74:12–49. doi: 10.3322/caac.21820, PMID: 38230766

[2] Leowattana W Leowattana P Leowattana T . Systemic treatment for metastatic colorectal cancer. World J Gastroenterol. (2023) 29:1569–88. doi: 10.3748/wjg.v29.i10.1569, PMID: 36970592 PMC10037252

[3] SEER program NIH National Cancer Institute . SEER cancer statistics review: colorectal cancer—5-year relative survival by stage. Surveillance, Epidemiology, and End Results (SEER) Program, National Institutes of Health. (2024). Available at: https://seer.cancer.gov/statfacts/html/colorect.html.

[4] Kiran NS Yashaswini C Maheshwari R Bhattacharya S Prajapati BG . Advances in precision medicine approaches for colorectal cancer: from molecular profiling to targeted therapies. ACS Pharmacol Trans Sci. (2024) 7:967–90. doi: 10.1021/acsptsci.4c00008, PMID: 38633600 PMC11019743

[5] Doleschal B Petzer A Rumpold H . Current concepts of anti-EGFR targeting in metastatic colorectal cancer. Front Oncol. (2022) 12:1048166. doi: 10.3389/fonc.2022.1048166, PMID: 36465407 PMC9714621

[6] Global Burden of Disease 2019 Cancer Collaboration Kocarnik JM Compton K Dean FE Fu W Gaw BL . Cancer incidence, mortality, years of life lost, years lived with disability, and disability-adjusted life years for 29 cancer groups from 2010 to 2019: A systematic analysis for the global burden of disease study 2019. JAMA Oncol. (2022) 8:420. doi: 10.1001/jamaoncol.2021.6987, PMID: 34967848 PMC8719276

[7] Cendra AS Pekarek L Ospino LR Dbouk Y Chnaiker S Luengo A . Predictive and prognostic biomarkers of recurrence in locoregional colorectal cancer. J Cancer. (2025) 16:3024–39. doi: 10.7150/jca.111828, PMID: 40740242 PMC12305430

[8] Di Nicolantonio F Vitiello PP Marsoni S Siena S Tabernero J Trusolino L . Precision oncology in metastatic colorectal cancer — from biology to medicine. Nat Rev Clin Oncol. (2021) 18:506–25. doi: 10.1038/s41571-021-00495-z, PMID: 33864051

[9] Muradi Muhar A Velaro AJ Prananda AT Nugraha SE Halim P Syahputra RA . Precision medicine in colorectal cancer: genomics profiling and targeted treatment. Front Pharmacol. (2025) 16:1532971. doi: 10.3389/fphar.2025.1532971, PMID: 40083375 PMC11903709

[10] Baretti M Personeni N Destro A Santoro A Rimassa L . Emergence of KRAS-mutation in liver metastases after an anti-EGFR treatment in patient with colorectal cancer: Are we aware of the therapeutic impact of intratumor heterogeneity? Cancer Biol Ther. (2018) 19:659–63. doi: 10.1080/15384047.2018.1450117, PMID: 29580164 PMC6067891

[11] Lu X Li Y Li Y Zhang X Shi J Feng H . Prognostic and predictive biomarkers for anti-EGFR monoclonal antibody therapy in RAS wild-type metastatic colorectal cancer: a systematic review and meta-analysis. BMC Cancer. (2023) 23:1117. doi: 10.1186/s12885-023-11600-z, PMID: 37974093 PMC10655341

[12] Parseghian CM Sun R Woods M Napolitano S Lee HM Alshenaifi J . Resistance mechanisms to anti–epidermal growth factor receptor therapy in *RAS/RAF* wild-type colorectal cancer vary by regimen and line of therapy. J Clin Oncol. (2023) 41:460–71. doi: 10.1200/JCO.22.01423, PMID: 36351210 PMC9870238

[13] Leite LF Noronha MM De Menezes JSA Da Conceição LD Almeida LFC Cappellaro AP . Anti-EGFR therapy in metastatic colorectal cancer: identifying, tracking, and overcoming resistance. Cancers. (2025) 17:2804. doi: 10.3390/cancers17172804, PMID: 40940901 PMC12427569

[14] Desai SA Patel VP Bhosle KP Nagare SD Thombare KC . The tumor microenvironment: shaping cancer progression and treatment response. J Chemotherapy. (2025) 37:15–44. doi: 10.1080/1120009x.2023.2300224, PMID: 38179655

[15] Wang S Wang J Chen Z Luo J Guo W Sun L . Targeting M2-like tumor-associated macrophages is a potential therapeutic approach to overcome antitumor drug resistance. NPJ Precis Oncol. (2024) 8:31. doi: 10.1038/s41698-024-00522-z, PMID: 38341519 PMC10858952

[16] Liu Z . Single-cell RNA sequencing reveals immunosuppressive mechanisms of MDSCs in lung cancer. In: Shi Z Hashash AH Khan ZH , editors. BIO Web of Conferences, vol. 174 (2025). p. 01016. doi: 10.1051/bioconf/202517401016

[17] Shi Y Guo Z Wang Q Deng H . Prognostic value of tumor-infiltrating lymphocyte subtypes and microorganisms in triple-negative breast cancer. J Cancer Res Ther. (2024) 20:1983–90. doi: 10.4103/jcrt.jcrt_41_24, PMID: 39792407

[18] Yang Y Sun D Ma X Wang T Wu J . CD68- and CD163-positive tumor-associated macrophages in renal clear cell carcinoma. Sci Rep. (2025) 15:35138. doi: 10.1038/s41598-025-19003-9, PMID: 41062564 PMC12508087

[19] Zhang W Li S Zhang C Mu Z Chen K Xu Z . Tumor-infiltrating lymphocytes predict efficacy of immunotherapy in advanced non-small cell lung cancer: a single-center retrospective cohort study. Acta Oncol. (2023) 62:853–60. doi: 10.1080/0284186x.2023.2228991, PMID: 37377003

[20] Lin Y Song Y Zhang Y Li X Kan L Han S . New insights on anti-tumor immunity of CD8+ T cells: cancer stem cells, tumor immune microenvironment and immunotherapy. J Trans Med. (2025) 23:341. doi: 10.1186/s12967-025-06291-y, PMID: 40097979 PMC11912710

[21] Huang R Kang T Chen S . The role of tumor-associated macrophages in tumor immune evasion. J Cancer Res Clin Oncol. (2024) 150:238. doi: 10.1007/s00432-024-05777-4, PMID: 38713256 PMC11076352

[22] Saeed AF . Tumor-associated macrophages: polarization, immunoregulation, and immunotherapy. Cells. (2025) 14:741. doi: 10.3390/cells14100741, PMID: 40422244 PMC12110377

[23] Navasardyan I Bonavida B . Regulation of T cells in cancer by nitric oxide. Cells. (2021) 10:2655. doi: 10.3390/cells10102655, PMID: 34685635 PMC8534057

[24] Menjivar RE Nwosu ZC Du W Donahue KL Hong HS Espinoza C . Arginase 1 is a key driver of immune suppression in pancreatic cancer. eLife. (2023) 12:e80721. doi: 10.7554/eLife.80721, PMID: 36727849 PMC10260021

[25] Karadima E Chavakis T Alexaki VI . Arginine metabolism in myeloid cells in health and disease. Semin Immunopathology. (2025) 47:11. doi: 10.1007/s00281-025-01038-9, PMID: 39863828 PMC11762783

[26] Hernandez-Ainsa M Velamazan R Lanas A Carrera-Lasfuentes P Piazuelo E . Blood-cell-based inflammatory markers as a useful tool for early diagnosis in colorectal cancer. Front Med. (2022) 9:843074. doi: 10.3389/fmed.2022.843074, PMID: 35795635 PMC9252519

[27] Ba Z-C Zhu X-Q Li Z-G Li Y-Z . Development and validation of a prognostic immunoinflammatory index for patients with gastric cancer. World J Gastroenterol. (2024) 30:3059–75. doi: 10.3748/wjg.v30.i24.3059, PMID: 38983960 PMC11230058

[28] Rafa H Benkhelifa S AitYounes S Saoula H Belhadef S Belkhelfa M . All-trans retinoic acid modulates TLR4/NF-*κ*B signaling pathway targeting TNF-*α*and nitric oxide synthase 2 expression in colonic mucosa during ulcerative colitis and colitis associated cancer. Mediators Inflammation. (2017), 1–16. doi: 10.1155/2017/7353252, PMID: 28408791 PMC5376956

[29] Benkhelifa S Rafa H Belhadef S Ait-kaci H Medjeber O Belkhelfa M . Aberrant up-regulation of iNOS/NO system is correlated with an increased abundance of Foxp3+ cells and reduced effector/memory cell markers expression during colorectal cancer: immunomodulatory effects of cetuximab combined with chemotherapy. Inflammopharmacology. (2019) 27:685–700. doi: 10.1007/s10787-019-00566-9, PMID: 30680650

[30] Touil-Boukoffa C Bauvois B Sancéau J Hamrioui B Wietzerbin J . Production of nitric oxide (NO) in human hydatidosis: Relationship between nitrite production and interferon-γ levels. Biochimie. (1998) 80:739–44. doi: 10.1016/S0300-9084(99)80027-3, PMID: 9865496

[31] Corraliza IM Soler G Eichmann K Modolell M . Arginase induction by suppressors of nitric oxide synthesis (IL-4, IL-10 and PGE2) in murine bone-marrow-derived macrophages. Biochem Biophys Res Commun. (1995) 206:667–73. doi: 10.1006/bbrc.1995.1094, PMID: 7530004

[32] Krzystek-Korpacka M Szczęśniak-Sięga B Szczuka I Fortuna P Zawadzki M Kubiak A . L-arginine/nitric oxide pathway is altered in colorectal cancer and can be modulated by novel derivatives from Oxicam class of non-steroidal anti-inflammatory drugs. Cancers. (2020) 12:2594. doi: 10.3390/cancers12092594, PMID: 32932854 PMC7564351

[33] Du T Han J . Arginine metabolism and its potential in treatment of colorectal cancer. Front Cell Dev Biol. (2021) 9:658861. doi: 10.3389/fcell.2021.658861, PMID: 34095122 PMC8172978

[34] Gawiński C Michalski W Mróz A Wyrwicz L . Correlation between lymphocyte-to-monocyte ratio (LMR), neutrophil-to-lymphocyte ratio (NLR), platelet-to-lymphocyte ratio (PLR) and tumor-infiltrating lymphocytes (TILs) in left-sided colorectal cancer patients. Biology. (2022) 11:385. doi: 10.3390/biology11030385, PMID: 35336759 PMC8945266

[35] Li Q Geng S Luo H Wang W Mo Y-Q Luo Q . Signaling pathways involved in colorectal cancer: pathogenesis and targeted therapy. Signal Transduction Targeted Ther. (2024) 9:266. doi: 10.1038/s41392-024-01953-7, PMID: 39370455 PMC11456611

[36] Ricci J-E . Tumor-induced metabolic immunosuppression: Mechanisms and therapeutic targets. Cell Rep. (2025) 44:115206. doi: 10.1016/j.celrep.2024.115206, PMID: 39798090

[37] Giatromanolaki A Tsolou A Daridou E Kouroupi M Chlichlia K Koukourakis MI . iNOS expression by tumor-infiltrating lymphocytes, PD-L1 and prognosis in non-small-cell lung cancer. Cancers. (2020) 12:3276. doi: 10.3390/cancers12113276, PMID: 33167430 PMC7694334

[38] Feng T Xie F Lyu Y Yu P Chen B Yu J . The arginine metabolism and its deprivation in cancer therapy. Cancer Lett. (2025) 620:217680. doi: 10.1016/j.canlet.2025.217680, PMID: 40157492

[39] Grzybowski MM Uçal Y Muchowicz A Rejczak T Kikulska A Głuchowska KM . Metabolomic reprogramming of the tumor microenvironment by dual arginase inhibitor OATD-02 boosts anticancer immunity. Sci Rep. (2025) 15:18741. doi: 10.1038/s41598-025-03446-1, PMID: 40437024 PMC12119792

[40] Ma Z Lian J Yang M Wuyang J Zhao C Chen W . Overexpression of Arginase-1 is an indicator of poor prognosis in patients with colorectal cancer. Pathol - Res Pract. (2019) 215:152383. doi: 10.1016/j.prp.2019.03.012, PMID: 30890279

[41] Niu F Yu Y Li Z Ren Y Li Zi Ye Q . Arginase: An emerging and promising therapeutic target for cancer treatment. Biomedicine Pharmacotherapy. (2022) 149:112840. doi: 10.1016/j.biopha.2022.112840, PMID: 35316752

[42] Wang X Yuwen T Zhong Y Li Z-G Wang X-Y . A new method for predicting the prognosis of colorectal cancer patients through a combination of multiple tumor-associated macrophage markers at the invasive front. Heliyon. (2023) 9:e13211. doi: 10.1016/j.heliyon.2023.e13211, PMID: 36798759 PMC9925966

[43] Hofmann L Harasymczuk M Huber D Szczepanski MJ Dworacki G Whiteside TL . Arginase-1 in plasma-derived exosomes as marker of metastasis in patients with head and neck squamous cell carcinoma. Cancers. (2023) 15:5449. doi: 10.3390/cancers15225449, PMID: 38001706 PMC10670520

[44] Wang X Xiang H Toyoshima Y Shen W Shichi S Nakamoto H . Arginase-1 inhibition reduces migration ability and metastatic colonization of colon cancer cells. Cancer Metab. (2023) 11:1. doi: 10.1186/s40170-022-00301-z, PMID: 36639644 PMC9838026

[45] Li H Feng X Hu Y Wang J Huang C Yao X . Development of a prognostic model based on ferroptosis-related genes for colorectal cancer patients and exploration of the biological functions of NOS2 *in vivo* and *in vitro*. Front Oncol. (2023) 13:1133946. doi: 10.3389/fonc.2023.1133946, PMID: 37346068 PMC10280989

[46] Cunha PP Bargiela D Minogue E Krause LCM Barbieri L Brombach C . Infiltration of tumors is regulated by T cell–intrinsic nitric oxide synthesis. Cancer Immunol Res. (2023) 11:351–63. doi: 10.1158/2326-6066.CIR-22-0387, PMID: 36574610 PMC9975666

[47] Chen T . Unveiling the significance of inducible nitric oxide synthase: Its impact on cancer progression and clinical implications. Cancer Lett. (2024) 592:216931. doi: 10.1016/j.canlet.2024.216931, PMID: 38701892

[48] Thiele Orberg E Fan H Tam AJ Dejea CM Destefano Shields CE Wu S . The myeloid immune signature of enterotoxigenic Bacteroides fragilis-induced murine colon tumorigenesis. Mucosal Immunol. (2017) 10:421–33. doi: 10.1038/mi.2016.53, PMID: 27301879 PMC5159334

[49] Pan J Lin Y Liu X Zhang X Liang T Bai X . Harnessing amino acid pathways to influence myeloid cell function in tumor immunity. Mol Med. (2025) 31:44. doi: 10.1186/s10020-025-01099-4, PMID: 39905317 PMC11796060

[50] Coburn LA Singh K Asim M Barry DP Allaman MM Al-Greene NT . Loss of solute carrier family 7 member 2 exacerbates inflammation-associated colon tumorigenesis. Oncogene. (2019) 38:1067–79. doi: 10.1038/s41388-018-0492-9, PMID: 30202097 PMC6377304

[51] Baier J Gänsbauer M Giessler C Arnold H Muske M Schleicher U . Arginase impedes the resolution of colitis by altering the microbiome and metabolome. J Clin Invest. (2020) 130:5703–20. doi: 10.1172/JCI126923, PMID: 32721946 PMC7598089

[52] Sougiannis AT VanderVeen B Chatzistamou I Kubinak JL Nagarkatti M Fan D . Emodin reduces tumor burden by diminishing M2-like macrophages in colorectal cancer. Am J Physiology-Gastrointestinal Liver Physiol. (2022) 322:G383–95. doi: 10.1152/ajpgi.00303.2021, PMID: 35018819 PMC8897011

[53] Omadhika W Solikhah S Adrianto A Purwestri Y Paramita D . M2 macrophage prominently distributed in the rat’s colon of DMH-induced inflammation associated colorectal cancer. Asian Pacific J Cancer Prev. (2024) 25:1357–62. doi: 10.31557/APJCP.2024.25.4.1357, PMID: 38679997 PMC11162729

[54] Xue Q Yan Y Zhang R Xiong H . Regulation of iNOS on immune cells and its role in diseases. Int J Mol Sci. (2018) 19:3805. doi: 10.3390/ijms19123805, PMID: 30501075 PMC6320759

[55] Park JV Chandra R Cai L Ganguly D Li H Toombs JE . Tumor cells modulate macrophage phenotype in a novel *in vitro* co-culture model of the NSCLC tumor microenvironment. J Thorac Oncol. (2022) 17:1178–91. doi: 10.1016/j.jtho.2022.06.011, PMID: 35798240 PMC9529910

[56] Grzywa TM Sosnowska A Matryba P Rydzynska Z Jasinski M Nowis D . Myeloid cell-derived Arginase in cancer immune response. Front Immunol. (2020) 11:938. doi: 10.3389/fimmu.2020.00938, PMID: 32499785 PMC7242730

[57] Wu B Zhang B Li B Wu H Jiang M . Cold and hot tumors: from molecular mechanisms to targeted therapy. Signal Transduction Targeted Ther. (2024) 9:274. doi: 10.1038/s41392-024-01979-x, PMID: 39420203 PMC11491057

[58] Cheng L-C Chao Y-J Wang C-Y Phan NN Chen Y-L Wang T-W . Cancer-derived transforming growth factor-β Modulates tumor-associated macrophages in ampullary cancer. OncoTargets Ther. (2020) 13:7503–16. doi: 10.2147/ott.s246714, PMID: 32821120 PMC7423398

[59] Chen Z Lin S Liang F Hou Z Yang Y Huang H . The prognostic and therapeutic value of the tumor microenvironment and immune checkpoints in pancreatic neuroendocrine neoplasms. Sci Rep. (2024) 14:24669. doi: 10.1038/s41598-024-75882-4, PMID: 39433799 PMC11494001

[60] Xu J Gao Y Ding Y Feng Y Chen J Zhang S . Correlation between Tregs and ICOS-induced M2 macrophages polarization in colorectal cancer progression. Front Oncol. (2024) 14:1373820. doi: 10.3389/fonc.2024.1373820, PMID: 39104717 PMC11298335

[61] Wei Q Deng Y Yang Q Zhan A Wang L . The markers to delineate different phenotypes of macrophages related to metabolic disorders. Front Immunol. (2023) 14:1084636. doi: 10.3389/fimmu.2023.1084636, PMID: 36814909 PMC9940311

[62] Toledo B Zhu Chen L Paniagua-Sancho M Marchal JA Perán M Giovannetti E . Deciphering the performance of macrophages in tumour microenvironment: a call for precision immunotherapy. J Hematol Oncol. (2024) 17:44. doi: 10.1186/s13045-024-01559-0, PMID: 38863020 PMC11167803

[63] Murray PJ Allen JE Biswas SK Fisher EA Gilroy DW Goerdt S . Macrophage activation and polarization: nomenclature and experimental guidelines. Immunity. (2014) 41:14–20. doi: 10.1016/j.immuni.2014.06.008, PMID: 25035950 PMC4123412

[64] Pinto ML Rios E Durães C Ribeiro R MaChado JC Mantovani A . The two faces of tumor-associated macrophages and their clinical significance in colorectal cancer. Front Immunol. (2019) 10:1875. doi: 10.3389/fimmu.2019.01875, PMID: 31481956 PMC6710360

[65] Kasurinen J Hagström J Kaprio T Beilmann-Lehtonen I Haglund C Böckelman C . Tumor-associated CD3- and CD8-positive immune cells in colorectal cancer: The additional prognostic value of CD8^+^-to-CD3^+^ ratio remains debatable. Tumor Biol. (2022) 44:37–52. doi: 10.3233/tub-211571, PMID: 35404299

[66] Wei C Ma Y Wang M Wang S Yu W Dong S . Tumor-associated macrophage clusters linked to immunotherapy in a pan-cancer census. NPJ Precis Oncol. (2024) 8:176. doi: 10.1038/s41698-024-00660-4, PMID: 39117688 PMC11310399

[67] Yin W Lv J Yao Y Zhao Y He Z Wang Q . Elevations of monocyte and neutrophils, and higher levels of granulocyte colony-stimulating factor in peripheral blood in lung cancer patients. Thorac Cancer. (2021) 12:2680–90. doi: 10.1111/1759-7714.14103, PMID: 34498383 PMC8520797

[68] Zhai Y Wu J Tang C Huang B Bi Q Luo S . Characterization of blood inflammatory markers in patients with non-small cell lung cancer. Int J Clin Exp Pathol. (2024) 17:165–72. doi: 10.62347/IPTW9741, PMID: 38859920 PMC11162609

[69] Abudukelimu K Tuerxuntayi A Aierken A Keranmu R Wufuer D . Combined CA125, NSE, and multiple inflammatory indices for diagnosis of oral squamous cell carcinoma. Front Oncol. (2025) 15:1543055. doi: 10.3389/fonc.2025.1543055, PMID: 40458729 PMC12127425

[70] Garcia-Flores LA Dawid De Vera MT Pilo J Rego A Gomez-Casado G Arranz-Salas I . Increased neutrophil counts are associated with poor overall survival in patients with colorectal cancer: a five-year retrospective analysis. Front Immunol. (2024) 15:1415804. doi: 10.3389/fimmu.2024.1415804, PMID: 39376564 PMC11456424

[71] Ohashi K Nishito Y Fukuda H Sadahiro R Yoshida Y Watanabe S . Neutrophil-to-lymphocyte ratio is a prognostic factor reflecting immune condition of tumor microenvironment in squamous cell lung cancer. Sci Rep. (2024) 14:429. doi: 10.1038/s41598-023-50378-9, PMID: 38172491 PMC10764784

[72] Tan X Wang Y Yu Y Zheng R Li J Chen S . Neutrophil-to-lymphocyte ratio predicts a poor prognosis for penile cancer with an immunosuppressive tumor microenvironment. Front Immunol. (2025) 16:1568825. doi: 10.3389/fimmu.2025.1568825, PMID: 40308599 PMC12041217

[73] Jiang J Ma T Xi W Yang C Wu J Zhou C . Pre-treatment inflammatory biomarkers predict early treatment response and favorable survival in patients with metastatic colorectal cancer who underwent first line cetuximab plus chemotherapy. Cancer Manage Res. (2019) 11:8657–68. doi: 10.2147/CMAR.S211089, PMID: 31576170 PMC6767765

[74] Passardi A Azzali I Bittoni A Marisi G Rebuzzi F Molinari C . Inflammatory indices as prognostic markers in metastatic colorectal cancer patients treated with chemotherapy plus Bevacizumab. Ther Adv Med Oncol. (2023) 15:17588359231212184. doi: 10.1177/17588359231212184, PMID: 38107830 PMC10722949

[75] Karan C Yaren A Demirel BC Dogan T Ozdemir M Demiray AG . Pretreatment PLR is preferable to NLR and LMR as a predictor in locally advanced and metastatic bladder cancer. Cancer Diagnosis Prognosis. (2023) 3:706–15. doi: 10.21873/cdp.10275, PMID: 37927800 PMC10619568

[76] Lin Z Ma C Cao W Ning Z Tan G . Prognostic significance of NLR, PLR, LMR and tumor infiltrating T lymphocytes in patients undergoing surgical resection for Hilar cholangiocarcinoma. Front Oncol. (2022) 12:908907. doi: 10.3389/fonc.2022.908907, PMID: 35719959 PMC9203898

[77] Zergoun AA Braikia S Boubnider MW Bouzid K Touil-Boukoffa C . Prognostic value of pro-inflammatory markers at the preoperative stage in Algerian women with breast cancer. Forum Clin Oncol. (2023) 14:45–56. doi: 10.2478/fco-2023-0021

[78] Qiang Z Zhang H Jin S Yan C Li Z Tao L . The prognostic value of arginase-1 and glypican-3 expression levels in patients after surgical intrahepatic cholangiocarcinoma resection. World J Surg Oncol. (2021) 19:316. doi: 10.1186/s12957-021-02426-9, PMID: 34715880 PMC8556943

[79] Tran DN Rozen V Nguyen LTK Jung J-S Coghill LM Hunter MI . ARG1 is a potential prognostic marker in metastatic endometrial cancer. Reprod Sci. (2024) 31:1632–41. doi: 10.1007/s43032-024-01493-z, PMID: 38388922 PMC11648120

[80] Lin C Li Z Zhu X Zhou W Lu X Zheng J . Abnormal β-hydroxybutyrylation modification of ARG1 drives reprogramming of arginine metabolism to promote the progression of colorectal cancer. Advanced Sci. (2025) 12:e02402. doi: 10.1002/advs.202502402, PMID: 40641413 PMC12520464

[81] Xing C Zhao L Zou W Peng X Xing X-L Li J . NOS2 as a prognostic biomarker for early-onset colorectal cancer based on public data and clinical validation analysis. Sci Rep. (2025) 15:4300. doi: 10.1038/s41598-025-88966-6, PMID: 39905237 PMC11794712

[82] Tang Y Li Q Zhou Z Bai H Xiao N Xie J . Nitric oxide-based multi-synergistic nanomedicine: an emerging therapeutic for anticancer. J Nanobiotechnology. (2024) 22:674. doi: 10.1186/s12951-024-02929-z, PMID: 39497134 PMC11536969

[83] Wang J Deng S Cheng D Gu J Qin L Mao F . Engineered microparticles modulate arginine metabolism to repolarize tumor-associated macrophages for refractory colorectal cancer treatment. J Trans Med. (2024) 22:908. doi: 10.1186/s12967-024-05652-3, PMID: 39375706 PMC11457421

[84] Niu Y Yuan X Guo F Cao J Wang Y Zhao X . Correlation between NLR combined with PLR score and prognosis of hepatocellular carcinoma after liver transplantation. Int J Gen Med. (2024) 17:2445–53. doi: 10.2147/IJGM.S450585, PMID: 38826508 PMC11141585

[85] Yang D Li P Meng Z Hu X Huang Z Huang H . Combined pretreatment neutrophil-lymphocyte ratio and platelet-lymphocyte ratio predicts survival and prognosis in patients with non-metastatic nasopharyngeal carcinoma: a retrospective study. Sci Rep. (2024) 14:9898. doi: 10.1038/s41598-024-59131-2, PMID: 38688967 PMC11061272

