# Supplementary figures and images for: NOS2/ARG1 axis and immune cell ratios as promising prognostic and predictive biomarkers for Cetuximab combined with chemotherapy in wt-KRAS human colorectal cancer
Source: Front Immunol. 2026 Jan 7;16:1700487. doi: 10.3389/fimmu.2025.1700487 (PMC12819718; doi:10.3389/fimmu.2025.1700487)

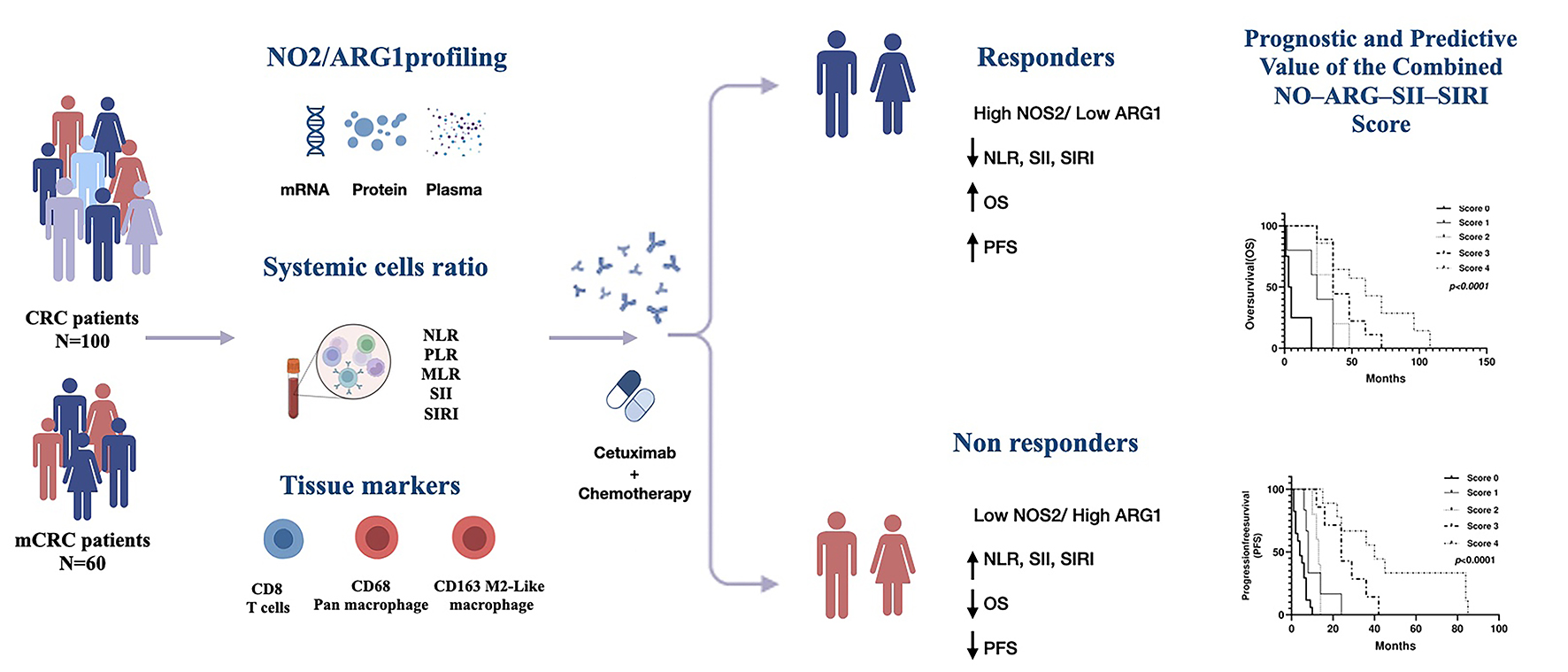

Supplement: Supplementary file 1 [file Image1.jpeg]

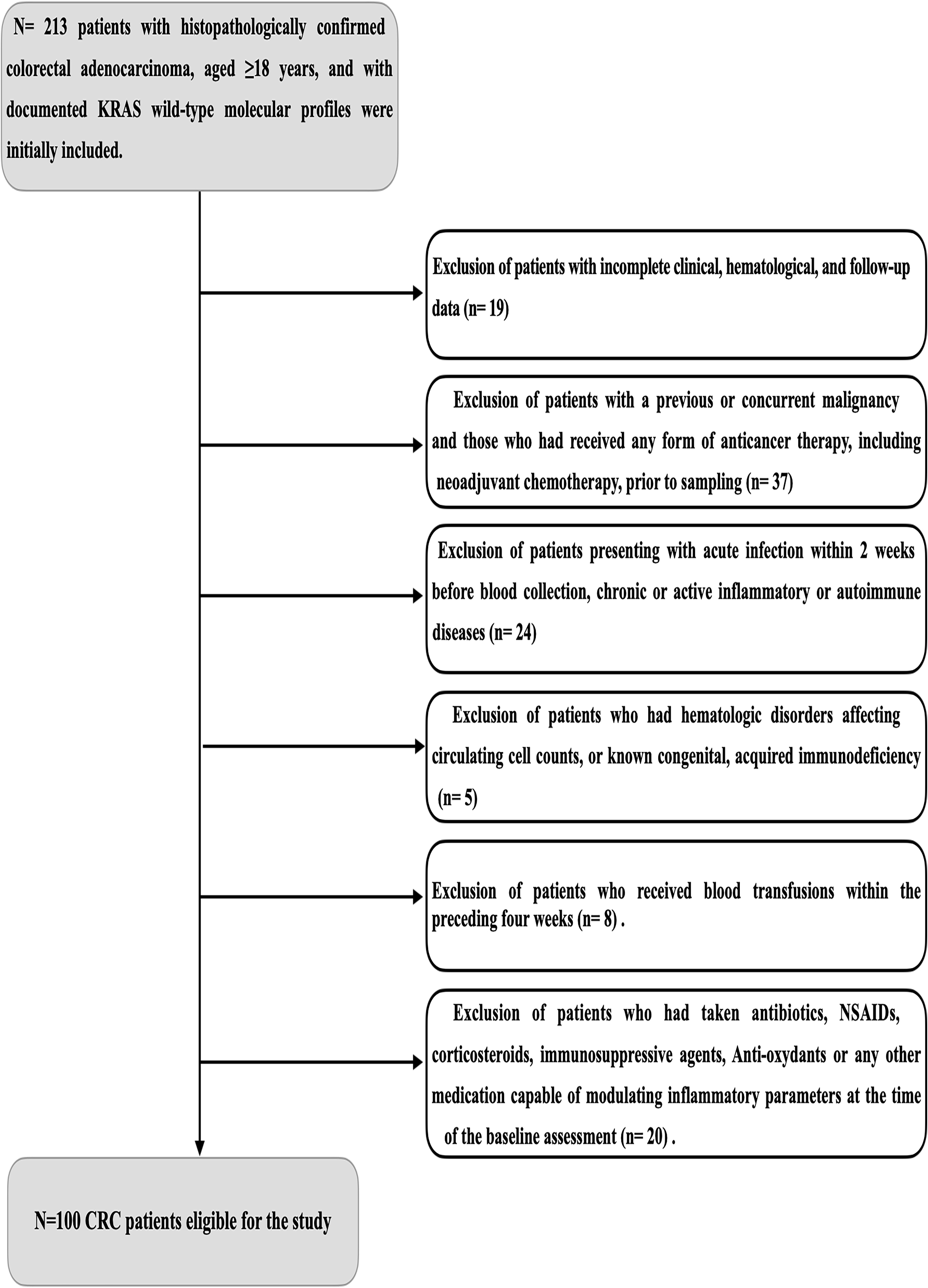

Supplement: Supplementary file 2 [file Image2.jpeg]
