# Supplementary material for: NOS2/ARG1 axis and immune cell ratios as promising prognostic and predictive biomarkers for Cetuximab combined with chemotherapy in wt-KRAS human colorectal cancer
Source: Front Immunol. 2026 Jan 7;16:1700487. doi: 10.3389/fimmu.2025.1700487 (PMC12819718; doi:10.3389/fimmu.2025.1700487)
Supplement: Supplementary file 3 [file Table1.docx]

**WRITTEN INFORMED CONSENT FORM FOR PATIENTS**

**Please read this consent form carefully before signing.**

**Do not hesitate to ask questions if any aspects seem unclear or if further clarification is needed.**

**This consent form is prepared in duplicate (2) copies, one (1) of which is to be given to the patient.**

**Place of performance:**

**Patient:                                                                     Sex:**

**Date of birth:                                                          Geographic origin:**

**Invitation to participate:** I declare that I have been informed, orally and in writing, in the simplest possible manner and in the language or dialect I understand best, of the objectives and procedures of the study, the presumed effects, the possible benefits and drawbacks, and the potential risk

I certify that I have read and understood the oral and written information provided to me regarding this study.

I have received satisfactory answers to the questions I asked regarding my participation in this study. I will keep the written patient information and receive a copy of the consent form.

I have been informed that I can continue all my previous treatment.

I have been informed that the medical tests included in the study are optional and that I can accept or refuse them without any impact on my treatment and follow-up care.

Benefits: I understand that my participation in this research will contribute to the advancement of research and knowledge in this field.

Confidentiality and anonymity: I understand that my personal data will be shared only in anonymous form.

Data storage: I have received assurances that the collected biological samples and results of the biological analyses will be stored securely.

Consent: I declare that I freely and willingly agree to participate in this research and that I have not been coerced into giving my consent.

**Place and date:                                                                               Patient's signature**
